# Genetic and antigenic variation of the bovine tick-borne pathogen *Theileria parva* in the Great Lakes region of Central Africa

**DOI:** 10.1186/s13071-019-3848-2

**Published:** 2019-12-16

**Authors:** Gaston S. Amzati, Appolinaire Djikeng, David O. Odongo, Herman Nimpaye, Kgomotso P. Sibeko, Jean-Berckmans B. Muhigwa, Maxime Madder, Nathalie Kirschvink, Tanguy Marcotty

**Affiliations:** 1 Research Unit of Veterinary Epidemiology and Biostatistics, Faculty of Agricultural and Environmental Sciences, Université Evangélique en Afrique, PO Box 3323, Bukavu, Democratic Republic of the Congo; 2 Unit of Integrated Veterinary Research, Department of Veterinary Medicine, Faculty of Sciences, Namur Research Institute for Life Sciences (NARILIS), University of Namur (UNamur), Rue de Bruxelles 61, 5000 Namur, Belgium; 3 Biosciences Eastern and Central Africa - International Livestock Research Institute (BecA-ILRI) Hub, PO Box 30709–00100, Nairobi, Kenya; 4 Present Address: Centre for Tropical Livestock Genetics and Health (CTLGH), The University of Edinburgh, Easter Bush, Midlothian, EH25 9RG Scotland, UK; 5 School of Biological Sciences, University of Nairobi, PO Box 30197–00100, Nairobi, Kenya; 6 Faculty of Medicine, University of Burundi, PO Box 1550, Bujumbura, Burundi; 7 Vector and Vector-Borne Disease Research Programme, Department of Veterinary Tropical Diseases, Faculty of Veterinary Science, University of Pretoria, P/Bag X04, Onderstepoort, Gauteng 0110 South Africa

**Keywords:** Tick-borne diseases, East Coast fever, Muguga cocktail vaccine, *Rhipicephalus appendiculatus*, Population genetics, Tp1, Tp2, Evolutionary dynamics, Agro-ecological zones

## Abstract

**Background:**

*Theileria parva* causes East Coast fever (ECF), one of the most economically important tick-borne diseases of cattle in sub-Saharan Africa. A live immunisation approach using the infection and treatment method (ITM) provides a strong long-term strain-restricted immunity. However, it typically induces a tick-transmissible carrier state in cattle and may lead to spread of antigenically distinct parasites. Thus, understanding the genetic composition of *T. parva* is needed prior to the use of the ITM vaccine in new areas. This study examined the sequence diversity and the evolutionary and biogeographical dynamics of *T. parva* within the African Great Lakes region to better understand the epidemiology of ECF and to assure vaccine safety. Genetic analyses were performed using sequences of two antigen-coding genes, *Tp1* and *Tp2*, generated among 119 *T. parva* samples collected from cattle in four agro-ecological zones of DRC and Burundi.

**Results:**

The results provided evidence of nucleotide and amino acid polymorphisms in both antigens, resulting in 11 and 10 distinct nucleotide alleles, that predicted 6 and 9 protein variants in *Tp1* and *Tp2*, respectively. *Theileria parva* samples showed high variation within populations and a moderate biogeographical sub-structuring due to the widespread major genotypes. The diversity was greater in samples from lowlands and midlands areas compared to those from highlands and other African countries. The evolutionary dynamics modelling revealed a signal of selective evolution which was not preferentially detected within the epitope-coding regions, suggesting that the observed polymorphism could be more related to gene flow rather than recent host immune-based selection. Most alleles isolated in the Great Lakes region were closely related to the components of the trivalent Muguga vaccine.

**Conclusions:**

Our findings suggest that the extensive sequence diversity of *T. parva* and its biogeographical distribution mainly depend on host migration and agro-ecological conditions driving tick population dynamics. Such patterns are likely to contribute to the epidemic and unstable endemic situations of ECF in the region. However, the fact that ubiquitous alleles are genetically similar to the components of the Muguga vaccine together with the limited geographical clustering may justify testing the existing trivalent vaccine for cross-immunity in the region.

## Background

*Theileria parva* is an intracellular apicomplexan parasite of cattle transmitted by the ixodid tick *Rhipicephalus appendiculatus*. The parasite infects and transforms bovine lymphocytes and causes an acute lymphoproliferative disease known as East Coast fever (ECF), which remains a major constraint to the improvement of cattle production in sub-Saharan Africa [1]. The disease is present in 12 countries including the Great Lakes region of Africa, where ticks are invading new areas through the extensive cross-border and seasonal movement of cattle for trade and pasture [2–6]. The geographical distribution of *T. parva* is mainly determined by that of its vector, for which predictive models have shown to have a wide range of suitable environments in Africa [7–9]. Thus, host dispersal and ecological traits affecting tick population dynamics and performance may drive the mechanisms for spreading various genotypes of the parasite in different agro-ecological zones (AEZs), which in turn could result in epidemics or disruption in the endemic stability of the disease in colonised areas [10–13].

Current management and control of ECF are achieved by limiting tick infestation through the use of acaricides, as well as by treatment of infected cattle with theilericidal drugs such as buparvaquone. However, the continuous use of acaricides is unsustainable, and treatment is only effective during the early stages of the disease [1]. In view of these limitations, vaccination remains the most effective control measure. The current approach to immunisation is the Infection and Treatment Method (ITM) of vaccination, which involves inoculation of live sporozoites from three parasite stocks known as “Muguga cocktail” and simultaneous treatment with a long-acting formulation of oxytetracycline [14]. The Muguga trivalent vaccine provides robust and long-lasting protection against challenge with homologous *T. parva* strains but limited protection against heterologous strains [15, 16]. It has been demonstrated that ITM vaccination induces strain-specific immunity, mediated by the major histocompatibility complex (MHC) class I-restricted CD8^+^ T cells killing *T. parva*-infected bovine host cells [17]. This suggests that the evolutionary dynamics of genetic diversity, which usually result in antigenic variation of parasites, enable *T. parva* to escape recognition by the host immune system [18, 19]. The genetic diversity of *T. parva* is thought to be driven by several mechanisms and factors, including gene isolation, genetic drift, mutation, host immunity and genetic exchange through recombination [18, 20]. Furthermore, the deployment of the Muguga cocktail in new areas can introduce new strains and establish locally persistent infections, a source of the permanent spread of the disease through transmission by local ticks, even in the absence of detectable parasitaemia [21–23]. The “foreign” vaccine strains may also recombine with local ones and produce new, potentially more virulent genotypes [20]. Thus, to reduce the risks of introducing foreign parasite strains, a comprehensive study of parasite genotypes circulating in the region is required prior to the deployment of a live vaccine.

Genetic studies using a panel of DNA mini- and microsatellite markers to characterise the extent of genotypic diversity of *T. parva* have been done across different African countries, including Tanzania [24, 25], Uganda [26–28], Kenya [29, 30], Zambia [31] and South Sudan [32]. These studies provided evidence of genetic exchange between some populations and a minimal genetic sub-structuring on a geographical basis. More recently, a number of antigen-coding genes and epitopes that are targets of bovine MHC-I restricted CD8^+^ T cells were identified in order to develop subunit vaccines against *T. parva* [33, 34]. Two of these reported antigens (*Tp1* and *Tp2*), which are immunodominant targets of bovine cytotoxic CD8^+^ T cells, were shown to be extensively polymorphic in parasite isolates from East Africa, especially in buffalo-derived *T. parva* and those from cattle co-grazing with buffalo [35–38]. The substantial variation previously reported in *Tp1* and *Tp2* antigens proved their great value for studying the antigenic composition and population structure of *T. parva.* In addition, antigenic variability in *T. parva* populations and immunodominance nature of the CD8+ T cell responses are believed to be major determinants of the parasite strain-restricted immunity, although the role of these antigens in immune protection conferred by the ITM vaccination is not clearly demonstrated yet [15–17, 19, 36, 39]. It can therefore be hypothesised that the movement of cattle carrying parasites or infected ticks and the agro-ecological variability could define the *T. parva* population structure through continuous introduction of new parasite variants that may affect the epidemiological landscape of ECF in the Great Lakes region of Africa. Thus, population genetic diversity studies of parasites are useful to better understand the epidemiology of ECF. In our recent genetic study of the tick vector, we identified two sympatric *R. appendiculatus* lineages, that strongly coexist in lowlands grazing areas in the Great Lakes region, where the climate is more arid than in the highlands [6]. The colonisation pattern of these two lineages in sympatric zones could result in different transmission dynamics and geographical distributions of *T. parva* genotypes.

Given the reported transboundary cattle movements and the evidence of agro-ecological conditions affecting the population structure of the tick vector in the Great Lakes region, the present study analysed: (i) the level of population genetic diversity and structure of *T. parva* parasites; (ii) their similitude with Muguga cocktail vaccine components; (iii) their phylogenetic relationships and biogeographical patterns; and (iv) their evolutionary dynamics. *Theileria parva* samples originating from three AEZs in the Democratic Republic of Congo and one AEZ in Burundi were analysed using the polymorphic antigens *Tp1* and *Tp2*, in comparison with published sequences, so as to further characterise the genetic relationships between *T. parva* genotypes evolving in different African countries. The knowledge of the population structure and evolution of *T. parva* should provide more insight for better understanding of the epidemiology of ECF and prediction of potential vaccine outcomes and breakthroughs for future sustainable management of ECF in the Great Lakes region.

## Methods

### Study area

**Fig. 1:**
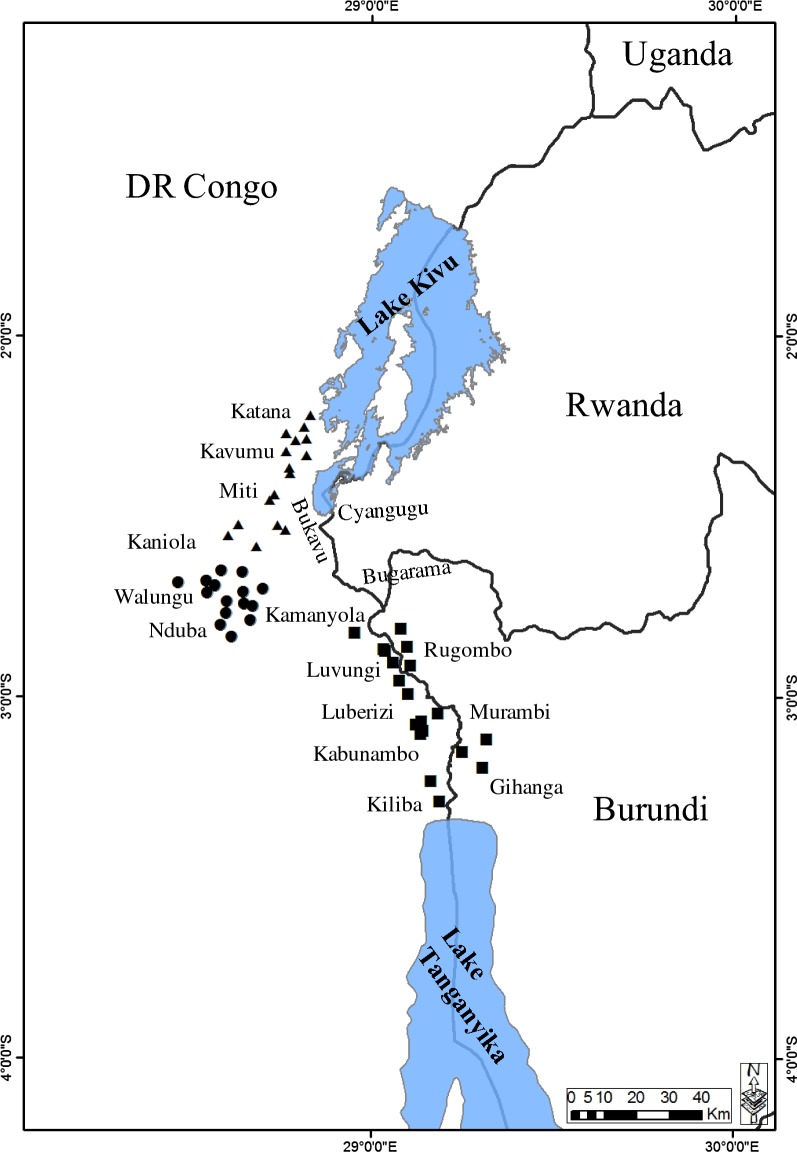
Map of the Great Lakes region showing sampling sites and their altitudes in the four agro-ecological zones of DRC and Burundi. Sampling sites located in the lowlands are indicated by squares (AEZ1: altitude < 1200 m), while circles indicate the sampling locations in midlands (AEZ2: altitude 1200–1800 m); and triangles denote sites located in the highlands (AEZ3: altitude 1600–2800 m)

The study was carried out in three AEZs of the South-Kivu Province of the Democratic Republic of Congo (DRC) and one AEZ in the Imbo valley of Burundi (Rugombo and Gihanga districts). Details of the geographical and climatic attributes of the AEZs and sampling sites characteristics were described in our earlier study [6] and mapped in Fig. 1. Briefly, the South-Kivu Province is covered by three main AEZs based on their altitudes: lowlands (< 1200 m: DRC AEZ1), midlands (1200–1800 m: DRC AEZ2) and highlands (1600–2800 m: DRC AEZ3), while the Imbo valley of Burundi falls into the lowlands area (< 1200 m: Burundi AEZ1). The rainfall period is bimodal and its duration varies significantly between AEZs. The annual rainfall increases with altitude while the temperature decreases. Majority of cattle found in the study area belong to indigenous Ankole breeds (Sanga type), raised mainly under a communal open grazing system and subjected to short and long-distance transhumance during the dry season. The livestock production system is characterised by irregular use of acaricides in all AEZs, except in more fenced commercial farms in the highlands where acaricides are usually applied on a weekly basis. No vaccination history against ECF had been reported in DRC, whereas a small-scale immunisation programme was introduced in Burundi between 1981 and 1987, using a cocktail vaccine of three local *T. parva* isolates (Gatumba, Gitega and Ngozi) for ITM vaccination [40].

### Cattle blood sample collection

A cross-sectional study was conducted during the late rainy season between February and April 2015, as previously described in Amzati et al. [6]. Blood samples were collected from indigenous cattle raised under traditional farming systems. Three cattle herds were randomly selected from 8–12 villages in each AEZ. In each herd, 5–10 adult cattle (over 3 years of age) were randomly sampled. The random function of Microsoft Excel program was used as random generator. Cattle blood samples were collected from the jugular vein into EDTA vacutainer tubes, then transferred to the laboratory in a cool box. On the same day, approximately 120 µl of each blood sample was spotted on Whatman FTA cards (Whatman Bioscience, Cambridge, UK), air-dried for 4 h at ambient temperature and stored individually in plastic bags containing dry silica gel packs at room temperature until used. A total of 480 blood samples were collected from the four AEZs (Additional file 1: Table S1).

### DNA isolation and PCR screening for *Theileria parva*

Genomic DNA (gDNA) was extracted from the dried blood spots using a commercial DNA extraction Kit (PureLink^®^ Genomic DNA Mini Kit, Invitrogen, Schwerte, Germany), according to the manufacturer’s instructions. DNA concentration was assessed using a Nanodrop spectrophotometer (Wilmington, Delaware, USA). Purified gDNA samples were screened for the presence of *T. parva* DNA using a nested PCR (nPCR) assay targeting the *T. parva-*specific conserved single-copy gene encoding the sporozoite microneme-rhoptry surface antigen, p104-kDa (GenBank: M29954) [41, 42]. Oligonucleotide outer primers IL3231 and IL755 and inner primers were IL4243 and IL3232 were used as previously described (Table 1) [22, 41] to amplify a 277-bp fragment of the p104 antigen gene. Amplification was performed using lyophilized AccuPower^®^ PCR Pre-mixes (Bioneer, Seoul, South Korea) for both primary and secondary PCR. The primary PCR reaction, in addition to AccuPower^®^ PCR Pre-mixes, contained 0.25 µM of each forward and reverse primers, 20 ng of gDNA, and nuclease-free distilled water added to bring the reaction to a final volume of 20 μl. The reaction mixture for the secondary amplification was as described for the primary PCR, except that the template was 1 μl of 3× diluted primary PCR products. A clean FTA punch was used as negative control for the DNA extraction and the positive control was a *T. parva* Muguga (F100 TpM) DNA obtained from BecA-ILRI Hub. The cycling conditions for the p104 gene have previously been described [22, 41], except for some minor modifications (Table 1). Six microliters of the secondary PCR products were analysed by electrophoresis in 1.8% agarose gels stained with GelRed (Biotium Inc., Hayward, USA) and visualised under UV light.

**Table 1:** PCR oligonucleotide primers used for amplification of p104, *Tp1* and *Tp2* genes with their corresponding annealing temperatures and amplicon sizes

Gene locus	Primer name	Primer sequence (5′–3′)	Annealing temperature (°C)	Amplicon size (bp)	Reference
p104	IL3231 (Fw1)	ATTTAAGGAACCTGACGTGACTGC	60	496	[22]
	IL755 (Rev1)	TAAGATGCCGACTATTAATGACACC			
	IL4243 (Fw2)	GGCCAAGGTCTCCTTCAGAATACG	55	277	[41]
	IL3232 (Rev2)	TGGGTGTGTTTCCTCGTCATCTGC			
*Tp1*	*Tp1*-Fw1	ATGGCCACTTCAATTGCATTTGCC	50	432	[36]
	*Tp1*-Rev1	TTAAATGAAATATTTATGAGCTTC			
	*Tp1*-Fw2	TGCATTTGCCGCTGATCCTGGATTCTG	55	405	[24, 37]
	*Tp1*-Rev2	TGAGCTTCGTATACACCCTCGTATTCG			
*Tp2*	*Tp2*-Fw1	ATGAAATTGGCCGCCAGATTA	50	525	[36]
	*Tp*2-Rev1	CTATGAAGTGCCGGAGGCTTC			
	*Tp2*-Fw2	ATTAGCCTTTACTTTATTATTTWCATTYTAC	54	504	[24, 37]
	*Tp2*-Rev2	CTATGAAGTGCCGGAGGCTTCTCCT			

*Abbreviations*: Fw1, forward outer; Fw2, forward inner; Rev1, reverse outer; Rev2, reverse inner

### PCR amplification and sequencing of *Tp1* and *Tp2* gene loci

Samples which tested positive for *T. parva* by an indication of the amplification of the p104 marker were used for the analysis of genetic diversity of *T. parva* samples. Two *T. parva* genes, *Tp1* and *Tp2*, were amplified using a previously described nested PCR [36, 37]. The sizes of amplified regions containing known CD8^+^ T cell epitopes were 405 bp and 504 bp for *Tp1* and *Tp2* nested amplicons, respectively [33, 34, 36]. Specific outer and inner primers used to amplify the two genes are presented in Table 1. The amplification was performed in a 20 μl PCR reaction, with the same components described for p104. Thermal cycling conditions for primary and secondary PCR for *Tp1* and *Tp2* genes were as described in Table 1. Six microliters of *Tp1* and *Tp2* nPCR products were analysed by electrophoresis in a 1.8% agarose gel. Amplicons obtained from the nPCR were purified using the QIAquick^®^ PCR Purification Kit (Qiagen GmbH, Hilden, Germany) following the manufacturer’s instructions and sequenced directly using inner forward and reverse primers on an Applied Biosystems ABI 3730 sequencer (Macrogen Inc. Europe, Amsterdam, The Netherlands).

### Prediction of amino acid sequences and epitope identification

Nucleotide sequences of *Tp1* and *Tp2* genes were manually edited, assembled and their consensus translated into amino acid sequences using CLC Main Workbench software v7.9.1 and the online translation tool EMBOSS-Transeq (https://www.ebi.ac.uk/Tools/st/emboss_transeq). A BLAST search was then performed to confirm species and gene identity of these sequences against available sequences in the GenBank database. The nucleotide and deduced amino acid sequences were aligned separately for each gene with corresponding Muguga reference sequences, one of the three components of the Muguga cocktail live vaccine (GenBank: JF451936 and JF451856 for *Tp1* and *Tp2*, respectively). Multiple nucleotide sequence alignments were constructed with a codon-based approach under the Muscle algorithm as implemented in the Translator X online platform (http://www.translatorx.co.uk) [43]. All alignments were visualised and inspected in the CLC Main Workbench software. Single nucleotide polymorphisms (SNPs), indels and sequence variants were then identified by comparing the generated consensus sequence for each sample to the corresponding Muguga sequence. The CD8^+^ T cell epitope regions and variants were identified using previously described positions presented in Additional file 2: Table S2 [34, 36].

### Population genetic analysis and phylogenetic reconstruction

*Tp1* and *Tp2* nucleotide sequences were collapsed into alleles using DnaSP software v6.11.01 [44]. Genetic diversity indices, including the number of segregating sites (*S*), nucleotide diversity (*π*), number of distinct alleles (*N*_*A*_), were estimated for each AEZ and for the entire dataset using the same software. In addition, the nucleotide diversity was estimated throughout the sequenced fragment of the *Tp2* gene based on a sliding-window of 100 nucleotides with a step size of 25 bp, to estimate the stepwise diversity across epitope coding regions. In order to assess the genetic structure within and between populations, pairwise estimates of genetic differentiation among populations based on Wright’s fixation index (*F*_*ST*_) and analysis of molecular variance (AMOVA) were implemented in Arlequin v3.5.2.2 [45]. The genetic differentiation (*F*_*ST*_) was interpreted as low (0–0.05), intermediate (0.05–0.15), great (0.15–0.25) and very great (*F*_*ST*_ < 0.25) [46]. The evolutionary divergence between gene alleles was estimated using proportion genetic distance (p-distance) in MEGA v7.0 [47].

Phylogenetic reconstructions were performed to further investigate the population structure and relationships among *T. parva* alleles in sub-Saharan Africa. Both representative *Tp1* and *Tp2* allele sequences found in the present study and those previously published, obtained from cattle and African buffalo (*Syncerus caffer*) across Africa, were used in the phylogenetic analyses and population differentiation. Published sequences comprised samples from Kenya (cattle-derived, buffalo-derived and buffalo-associated parasites), South Sudan and laboratory isolates from Kenya, Uganda, Tanzania, Zambia and Zimbabwe. The *Tp1* and *Tp2* gene sequences of the three *T. parva* stocks used in the live trivalent Muguga vaccine (Muguga, Serengeti-transformed and Kiambu-5) were also included in the analyses. The obtained overall representative allele datasets were aligned for each gene based on their corresponding amino acid translations using the Translator X server with its Muscle algorithm. Sites that were ambiguously aligned were eliminated from the protein alignment before back-translate to nucleotides using the GBlocks program with default parameters. In addition, the *Tp1* and *Tp2* alignments for individual sequences generated in the present study (from the Great Lakes region) were visually checked and concatenated to generate a data matrix (*Tp1* + *Tp2*) in order to maximise the phylogenetic signal. Samples with missing data for one locus were excluded from the concatenated dataset. Phylogenetic reconstructions were then performed for the *Tp1* and *Tp2* representative gene alleles separately, as well as for the concatenated nucleotide matrix in MEGA software using the Neighbor-Joining (NJ) algorithm by performing 1000 bootstrap replications. The best-fitting nucleotide substitution model for each dataset was estimated under the Bayesian information criterion (BIC) using MEGA. The evolutionary distances for each of the three datasets were computed using the Tamura 3-parameter (T92) model of nucleotide sequence evolution in which rate variation among sites was modelled according to gamma distribution. The phylogenetic trees were rooted with orthologous sequences from *Theileria annulata* as the outgroups (GenBank: TA17450 for *Tp1* and TA19865 for *Tp2*). Furthermore, a median-joining (MJ) network was constructed using cattle-derived *Tp2* nucleotide sequences generated during this study as well as published sequences, to investigate the ancestral relationships among *T. parva* alleles on the basis of their geographical origins. The network was computed through the default MJ algorithm described by Bandelt et al. [48] in the PopArt software [49]. Invariant sites were removed from the dataset for network reconstruction.

### Molecular evolutionary dynamics

*Theileria parva* demographic dynamics were analysed using selective neutrality statistics Fu and Liʼs D* and F* [50] and Tajima’s *D* [51] to evaluate the departure from neutral evolution or evidence of natural selection constraint for each studied population as implemented in the DnaSP and Arlequin software. To further assess the selective constraint within *Tp2* epitope coding regions, a sliding-window was estimated for the overall data set within a window of 100 bp using a step size of 25 bp. The significance of these statistics was tested with a coalescence-based approach using 1000 simulations. Statistically significant positive values of neutrality tests indicate an excess of intermediate-frequency alleles in the population than expected, that could be due to balancing selection, population structure or bottlenecks, while negative values denote an excess of rare polymorphisms in a population, which provides evidence of purifying, directional (positive) selection or population expansion. Population dynamics were further assessed with mismatch distribution of pairwise nucleotide differences between sequences in the Arlequin software.

## Results

### PCR amplification and gene polymorphisms

Of the 480 samples investigated, 119 produced a p104 amplicon suggesting that they contained *T. parva* DNA. These were subjected to *Tp1* and *Tp2* amplification and sequencing; sequences were successfully generated from 116 and 96 samples, respectively (Additional file 3: Table S3). We were unable to obtain amplicons or sequences from 3 samples for the *Tp1* gene and 23 samples for the *Tp2* gene. Novel *Tp1* and *Tp2* sequences were submitted and are available in the GenBank under accession numbers: MF449288-MF449294 for *Tp1*; and MF449295-MF449302 for *Tp2*. The 405-bp sequence region of *Tp1* encodes 134 amino acids (25% of the 543 amino acids of the full-length *Tp1* gene). This region is located between nucleotides 537 and 941 of the reference genome of the *T. parva* Muguga strain (GenBank: XP_762973), while the 504-bp region of the *Tp2* gene encodes 167 amino acids of the 174 amino acid-long protein encoded by the reference *T. parva* Muguga genome (GenBank: XP_765583). Sequence analyses showed moderate synonymous and nonsynonymous nucleotide substitutions randomly distributed along the *Tp1* sequence, including in the single CD8+ T cell target epitope, as well as an indel of 12 nucleotide insertion (TCT GCA CCT CCT) corresponding to the 4 amino acid residues SAPP. In contrast, the analyses revealed extensive polymorphisms in the *Tp2* gene, both at the nucleotide and amino acid levels, which were also identified within the six epitope regions. To further understand the phylogenetic relationships between *T. parva* parasites in sub-Saharan Africa, a comprehensive population genetic analysis was conducted, including *Tp1* and *Tp2* sequences retrieved from the GenBank.

### Sequence diversity in the *Tp1* gene locus

Sequence analysis of *Tp1* gene fragment detected 11 distinct alleles in the 116 sequenced DNA samples (Table 2, Additional file 4: Figure S1). These alleles were defined by 14 single-nucleotide polymorphisms (SNPs) and one in-frame indel insertion of 12 nucleotides compared with the reference *T. parva* Muguga genome sequence (identical to Serengeti and Kiambu-5 sequences for *Tp1*). The insertion occurred in four samples with the *Tp1* sequences identical to that of allele 45, which is genetically the most distant from the Muguga reference sequence (Additional file 5: Table S4). On the other hand, *Tp1* allele 1 (present in the three *T. parva* stocks components of the trivalent Muguga vaccine) was the most predominant allele, identified in 76 (65.5%) of the 116 samples. The overall nucleotide polymorphism in the *Tp1* gene was π = 0.5%. The lowest genetic diversity was obtained in DRC AEZ3, where all the 25 sequences were represented by the *T. parva* Muguga allele 1. The three other AEZs (DRC AEZ1, 2 and Burundi AEZ1) had very similar levels of nucleotide diversity (Table 2).

**Table 2:** *Tp1* and *Tp2* sequence diversity in cattle-derived *T. parva* from RDC and Burundi

Gene locus	AEZ	Sample size	Nucleotide sequences			Amino acid sequences	
			Polymorphic sites^a^	No. of alleles^b^	Nucleotide diversity ± SD	Polymorphic sites^a^	No. of antigen variants^b^
*Tp1*	DRC AEZ1	31	12 + ind	7	0.008 ± 0.002	7 + ind	5
	DRC AEZ2	27	12 + ind	8	0.007 ± 0.002	7 + ind	6
	DRC AEZ3	25	0	1	0	0	1
	Burundi AEZ1	33	4	3	0.005 ± 0.0004	3	3
Overall *Tp1*		116	14 + ind	11	0.005 ± 0.0007	7 + ind	6
*Tp2*	DRC AEZ1	25	166	7	0.13 ± 0.025	82	7
	DRC AEZ2	20	165	5	0.17 ± 0.016	81	5
	DRC AEZ3	23	2	2	0.002 ± 0.0004	2	2
	Burundi AEZ1	28	175	6	0.16 ± 0.017	85	5
Overall *Tp2*		96	181	10	0.14 ± 0.01	88	9

^a^The insertion region (ind) was excluded for the determination of the number of polymorphic sites and the nucleotide diversity

^b^Alleles represent distinct nucleotide sequences diverged at least by one substitution (Additional file 4: Figure S1, Additional file 8: Figure S2), while antigen variants represent predicted distinct protein sequences (Figs. 2, 4)

*Abbreviations*: AEZ, agro-ecological zone; ind, indels; SD, standard deviation

**Fig. 2:**
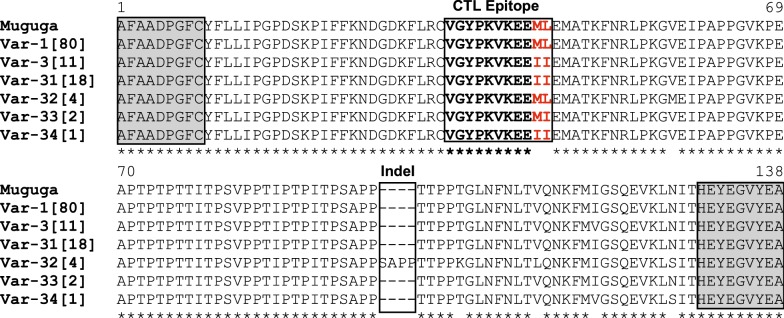
Multiple amino acid sequence alignment of six *Tp1* antigen variants in 116 *T. parva* samples obtained from DRC and Burundi. Antigen variants are named var-1 to var-34. The single letter amino acid code is used. The antigen variants nomenclature used in this study was first proposed by Pelle et al. [36]. Variants var-1 and var-3 were first described by Pelle et al. [36] and var-31 by Salih et al. [37]. The numbers in square brackets behind variants names indicate the number of *T. parva* isolates represented by each variant. The single previously identified *T. parva* CD8^+^ T cell target epitope is bolded and boxed. The polymorphic residues in the T cell epitope are coloured in red. Conserved amino acid residues are denoted by (*) below the alignment, and dashes (–) denote insertion region. Nested PCR primers used for sequencing are shaded and boxed in flanked regions. The Muguga sequence (GenBank: JF451936) was used as the reference sequence; it represents the other component of the Muguga cocktail vaccine (Serengeti-transformed and Kiambu 5) that are identical to Muguga strain sequence for the *Tp1* locus. *Tp1* antigen variant var-1 is found in the three Muguga vaccine strains. Corresponding gene alleles and sample characteristics are presented in Additional file 3: Table S3

Moreover, the 11 *Tp1* alleles allowed to predict six distinct antigen variants, distinguished by amino acid changes at seven polymorphic residues and one insertion motif of four amino acids (that contrasted with 92% of conserved amino acid residues) (Fig. 2, Table 2). The most common antigen variant (var1), which is present in the *T. parva* strains Muguga, Serengeti and Kiambu-5, was found in 69% (80/116 samples) of samples obtained from all AEZs. Furthermore, antigen variants 3 and 31, with the smallest genetic distances to the variant 1, accounted for 10% and 16% of the total *T. parva* samples, respectively; while variants 32–34 were rarely present and were only observed in DRC AEZ1 and 2. In most cases, the predicted protein variants of gene alleles containing unique sequence were identical or nearly similar to the most common antigens due to synonymous substitutions (Additional file 4: Figure S1, Additional file 6: Table S5).

The multiple alignment of predicted *Tp1* amino acid sequences revealed the presence of three different CD8^+^ T cell epitope variants, observed in the defined single *Tp1* CD8^+^ T cell epitope region (VGYPKVKEEML) (Fig. 2, Additional file 2: Table S2). The detailed geographical distribution of epitope variants is presented in Additional file 7: Table S6. Briefly, the epitope variant ending with -ML (which is also present in the three *T. parva* Muguga cocktail vaccine stocks) was found in the majority of samples from all four AEZs (72%: 84 out of 116 samples), followed by the epitope variant -II which was observed in 30 samples (26%) and particularly absent in *T. parva* samples from the highlands of DRC (DRC AEZ3). The third epitope variant (-MI) was only present in two samples from DRC AEZ1 and 2. We noticed the abundance of two of the three epitope variants in Burundi AEZ1, where approximately half of *T. parva* samples carried the epitope-II.

### Sequence diversity in the *Tp2* gene locus

**Fig. 3:**
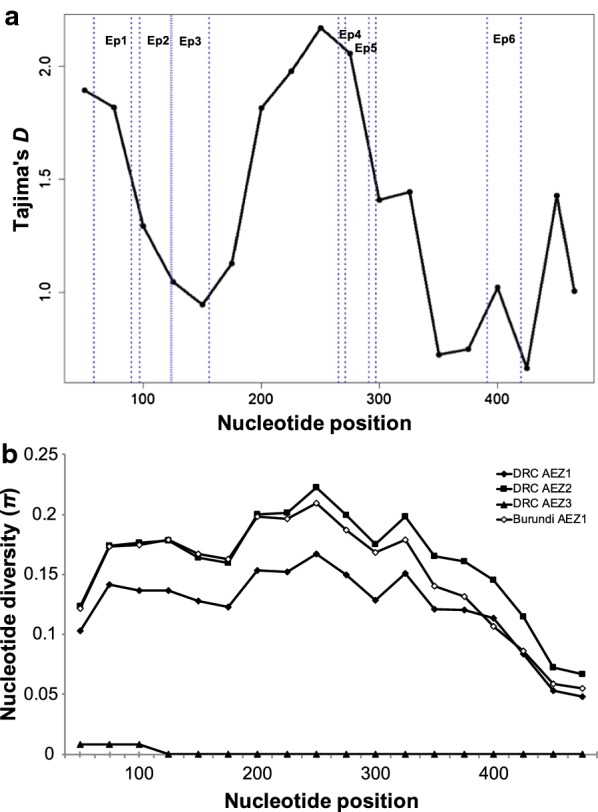
*Tp2*-based sliding-window plot of Tajima’s *D* statistics (**a**) and nucleotide diversity (**b**) of *T. parva* sequences from the Great Lakes region. A window length of 100 nucleotides and a step size of 25 bp were used. The maximum nucleotide diversity and Tajima’s *D* values are observed between the nucleotide positions 200 and 300, containing the *Tp2* epitopes 4 and 5. *Abbreviation*: Ep1-6, epitope1-6

A total of 10 unique *Tp2* alleles were identified among the 96 sequenced *T. parva* samples (Table 2, Additional file 8: Figure S2). The 10 alleles were determined by SNPs detected at 181 of the 504 nucleotide positions (variation in 36% of the nucleotide residues). The majority of variable sites (179 of 181) were parsimony informative and there were no deletions or insertions in the *Tp2* sequences analysed. The overall nucleotide polymorphism (π) was 14%, with the highest level of DNA diversity observed among *T. parva* samples obtained from cattle raised in DRC AEZ2 and Burundi AEZ1. In contrast, the nucleotide diversity was lower in DRC AEZ3 where only two *Tp2* alleles (alleles 1 and 2) were described (Table 2). The sliding-window plot revealed that samples from different AEZs shared similar patterns of diversity through their sequences, with the highest polymorphism observed between nucleotide positions 200–300 in most populations, except in DRC AEZ3 were the diversity was found between positions 50 and 100 (Fig. 3a, b). The number of alleles varied from five to seven among the *T. parva* samples in DRC (AEZ1 and 2) and Burundi AEZ1 (Table 2). The *Tp2* allele 1 (which is the Muguga and Serengeti allele) was the most ubiquitous, being observed in 39 of the 96 samples (41%) (Additional file 6: Table S5, Additional file 8: Figure S2). The next most common *Tp2* allele in the region was allele 2 (the *T. parva* Kiambu-5 allele, a component of the trivalent Muguga cocktail vaccine), and was present in 22 samples (23%). The majority of *T. parva* samples (61 of the 96 samples; 64%) were similar to the three component stocks of the Muguga cocktail, as observed for *Tp1* alleles. Interestingly, although the Muguga/Serengeti type was the most present in the region, it was less abundant than the Kiambu-5 type in Burundi AEZ1. In addition, alleles of the three components of the live vaccine were found in only 6 out of the 20 samples (30%) from DRC AEZ2. Moreover, the most common *Tp2* alleles in this DRC AEZ2 (alleles 56 and 57) were genetically the most distant from Muguga/Serengeti and Kiambu-5 alleles (p-distance > 25%) (Additional file 5: Table S4).

**Fig. 4:**
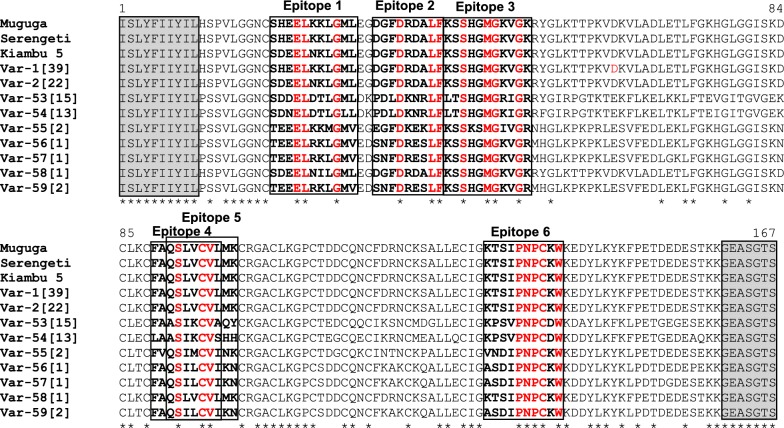
Multiple amino acid sequence alignment of nine *Tp2* antigen variants detected in 96 *T. parva* samples from DRC and Burundi. Amino acids are denoted by the single-letter codes. Var-1 to var-59 are variant names. The antigen variants nomenclature used in this study was first initiated by Pelle et al. [36]. Antigen variants var-1 and var-2 were described in Pelle et al. [36] and Salih et al. [37] and are, respectively, Muguga (identical to Serengeti-transformed) and Kiambu-5 strains. Reference sequences component of the Muguga cocktail live vaccine are represented by Muguga (GenBank: JF451856), Serengeti (Serengeti-transformed, GenBank: JF451862) and Kiambu-5 (GenBank: JF451880). The numbers in square brackets behind variants names indicate the number of *T. parva* samples represented by each variant. The six previously described epitopes (epitope1-6), that are the target of the bovine CD8^+^ T cells immune responses are bolded and boxed. The conserved amino acid residues in the epitopes are coloured in red. The star (*) below the alignment indicates positions of conserved amino acid residues. The shaded and boxed flanked regions denote the inner primers used for sequencing. *Tp2* Antigen variants var-1 and var-2 are found in Muguga/Serengeti and Kiambu-5 strains, respectively. Corresponding gene alleles and sample characteristics are presented in Additional file 3: Table S3

The nucleotide variation observed in the 10 *Tp2* alleles resulted in nine distinct protein variants with variations found at 88 amino acid residue positions (53% of variation) (Fig. 4). The results of antigenic variability of the *Tp2* gene are summarised in Table 2. In general, the number of antigen variants found in *T. parva* samples from different AEZs remained the same as for *Tp2* gene alleles, except in Burundi AEZ1 where the nucleotide substitution observed in allele 59 was synonymous and therefore this allele together with allele 57 were translated into protein variant 54 (Additional file 6: Table S5). These results reveal that most of SNPs found in the *Tp2* gene were non-synonymous, increasing the antigenic variability for the overall data set.

Furthermore, multiple alignment of *Tp2* amino acid sequences revealed an extensive degree of polymorphism in the six defined CD8^+^ T cell epitopes within the sequenced gene region (Table 3, Additional file 2: Table S2). The numbers of epitope variants ranged from four for epitope 2 to seven for epitope 1 (Table 3, Fig. 4). Within these epitopes, the number of conserved residues varied from three to five amino acid positions. Epitope 6 had the highest number of conserved amino acid residues, with residues 135–138 found in all protein sequences. Two variants (SHEELKKLGML and SDEELNKLGML) out of the seven variants of epitope 1 were identical to Muguga cocktail vaccine stock variants (Muguga/Serengeti and Kiambu-5) and comprised together 61 out of the 96 *Tp2* sequences studied (64%) (Table 3, Additional file 7: Table S6). The majority of sequences described in the present study carried the Muguga/Serengeti epitope variants in most AEZs, except in Burundi AEZ1 where the Kiambu-5 variant was the most prevalent for epitope 1, and in DRC AEZ2 where the most prevalent epitope variants were not present in the Muguga cocktail (Additional file 7: Table S6). Strikingly, despite the divergence distribution of epitope variants in different AEZs, a large number of overall major variants were common to all AEZs.

**Table 3:** *Tp1* and *Tp2* CD8^+^ T cell epitope variants identified in cattle-derived *T. parva* from DRC and Burundi

*Tp1* epitope variants (Tp1_35–45_)	*Tp2* epitope variants					
	Epitope 1 (Tp2_20–30_)	Epitope 2 (Tp2_33–41_)	Epitope 3 (Tp2_42–52_)	Epitope 4 (Tp2_89–97_)	Epitope 5 (Tp2_91–99_)	Epitope 6 (Tp2_131–140_)
**VGYPKVKEEML** (var-1, 32)	**SHEELKKLGML** (var-1)	**DGFDRDALF** (var-1, 2, 58)	**KSSHGMGKVGK** (var-1, 2, 58)	**FAQSLVCVL** (var-1, 2, 58)	**QSLVCVLMK** (var-1, 2, 58)	**KTSIPNPCKW** (var-1, 2, 58)
VGYPKVKEEII (var-3, 31, 34)	*TEEELKKMGMV* (var-55)	*EGFDKEKLF* (var-55)	*KSSKSMGIVGR* (var-55)	*FVQSIMCVI* (var-55)	*QSIMCVINK* (var-55)	*VNDIPNPCKW* (var-55)
VGYPKVKEEMI (var-33)	*TEEELRKLGMV* (var-56, 57, 59)	*SNFDRESLF* (var-56, 57, 59)	KSSHGMGKVGR (var-56, 57, 59)	*FAQSILCVI* (var-56, 57, 59)	*QSILCVIKN* (var-56, 57, 59)	*ASDIPNPCKW* (var-56, 57, 59)
	SDNELDTLGLL (var-54)	PDLDKNRLF (var-53, 54)	LTSHGMGKIGR (var-54)	LAASIKCVS (var-54)	ASIKCVSHH (var-54)	KPSVPNPCDW (var-53, 54)
	SDDELDTLGML (var-53)		LTSHGMGRIGR (var-53)	FAASIKCVA (var-53)	ASIKCVAQY (var-53)	
	**SDEELNKLGML** (var-2)					
	*SDEELNILGML* (var-58)					

*Notes:* Epitope variants were identified using the reference amino acid positions presented in Additional file 2: Table S2. Numbers in brackets following the epitope sequences correspond to antigen variants carrying the epitopes (Figs. 2, 4). Epitope variants described for the first time are in italic and those found in the Muguga cocktail vaccine are in bold. *Tp2* antigen variants var-1 and var-2 are found in Muguga (identical with Serengeti-transformed) and Kiambu-5 strains, respectively. *Tp1* antigen variant var-1 is found in Muguga (identical with Serengeti-transformed and Kiambu-5)

*Abbreviations*: Var-1 to var-59, antigen variant names

### Phylogenetic and phylogeographical patterns of *T. parva* populations in sub-Saharan Africa

In order to elucidate the phylogeographical structure and the evolutionary relationships among *T. parva* allelic sequences, the nucleotide sequences generated from the present study were analysed together with previously sequenced *T. parva* isolates from cattle and buffalo from different sub-Saharan African countries (Kenya, South Sudan, Tanzania, Uganda, Zambia and Zimbabwe) and the three component stocks of the Muguga cocktail live vaccine obtained from GenBank. In total, 274 *Tp1* and 241 *Tp2* sequences were analysed (Additional file 9: Table S7, Additional file 10: Table S8). The allelic analysis yielded 48 distinct alleles for *Tp1* and 61 different *Tp2* alleles. Of these *T. parva* alleles identified in Africa, seven *Tp1* alleles (A43–A49) and eight *Tp2* alleles (A56-A63) were new and exclusive to the Great Lakes region of Central Africa. In addition, 22 *Tp1* alleles (A13–A34) and 36 *Tp2* (A06–A43) were exclusively found in buffalo-derived or buffalo-associated *T. parva* isolates. The phylogenetic tree constructed from *Tp1* gene alleles failed to provide strong phylogenetic signal (Additional file 11: Figure S3), whereas the one based on *Tp2* alleles showed that *T. parva* parasites are more clustered depending on their mammalian host species than their geographical sub-structuring.

**Fig. 5:**
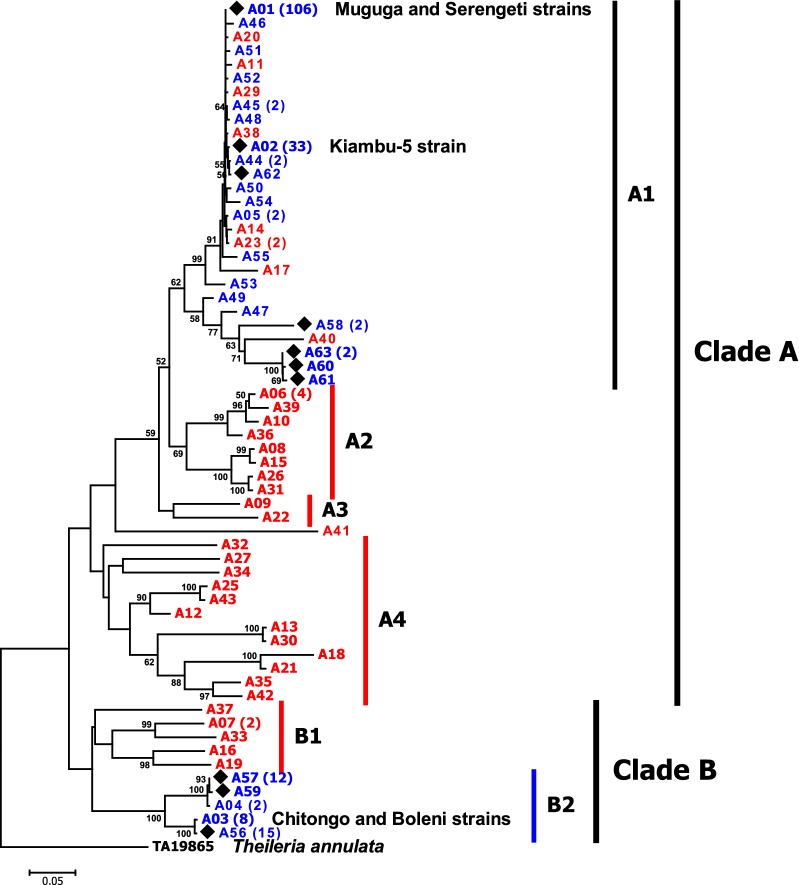
Neighbor-Joining tree showing phylogenetic relationships among the 61 *Tp2* gene alleles described in Africa (A01–A63). *Tp2* gene alleles obtained from cattle in the present study are indicated by black diamonds. *Theileria parva* alleles found in cattle with no association with buffalo and in laboratory stocks are coloured in blue, while buffalo-derived and buffalo-associated alleles are depicted in Red. Bootstrap values (> 50%) are shown above branches. The *Tp2* homologous sequence of *T. annulata* (GenBank: TA19865) was used as the outgroup. The numbers in brackets behind allele names denote the number of *T. parva* isolates carrying the allele. The detailed *Tp2* alleles distribution and their corresponding populations/AEZs are presented in Additional file 10: Table S8. *Tp2* allele A01 corresponds to isolates identical to Muguga and Serengeti-transformed strains, while *Tp2* allele A02 represents isolates identical to Kiambu-5 strain

The NJ phylogenetic tree performed on the 61 *Tp2* alleles (representative of 241 individual sequences) distinguished two main phylogenetic groups (clades A and B) (Fig. 5). The two main groups comprised four (A1–A4) and two (B1 and B2) sub-clades for clade A and clade B, respectively. The larger clade (clade A), containing the three component stocks of the Muguga cocktail vaccine, was composed of the majority of sequences. These sequences carried 20 *Tp2* alleles from cattle-derived *T. parva* (162 sequences of the 241 overall individual sequences; 67%) and 31 alleles from buffalo and cattle sharing grazing land with buffalo (35 individual sequences). The cattle-derived sequences found in this clade were clustered within the sub-clade A1 together with the three vaccine strains and were broadly distributed in various geographical areas in Africa (DRC AEZ1, 2 and 3, Burundi AEZ1, Kenya, South Sudan and Katete in the Eastern Province of Zambia) (Additional file 10: Table S8). In general, the more diverse buffalo-derived isolates found in clade A tend to be clustered in exclusive separate sub-clades (A2, A3 and A4), although sub-clade A1 contained mixed *T. parva* sequences from cattle and buffalo (or buffalo-associated cattle). The minor clade (clade B) contained 10 alleles (44 sequences) and had two independent sub-clades. The first sub-clade (B1) consisted exclusively of *T. parva* sequences from buffalo (6 sequences giving 5 alleles), while the second sub-clade (B2) comprised only cattle-derived *T. parva* (38 sequences carrying 5 alleles). Cattle-derived parasites found in clade B originated from DRC (AEZ1 and 2), Burundi AEZ1, Kenya, Uganda, Southern Province of Zambia (Chitongo) and Zimbabwe (Boleni). It is worth noting that cattle-derived samples from the lowlands and midlands of DRC and Burundi contained more diverse *T. parva* alleles which were consistently found in the two main *Tp2* clades and had no obvious association between allelic clades or sub-clades and their geographical origins. However, remarkably all the *T. parva* samples from the highlands (DRC AEZ3) were clustered within the major clade (clade A).

**Fig. 6:**
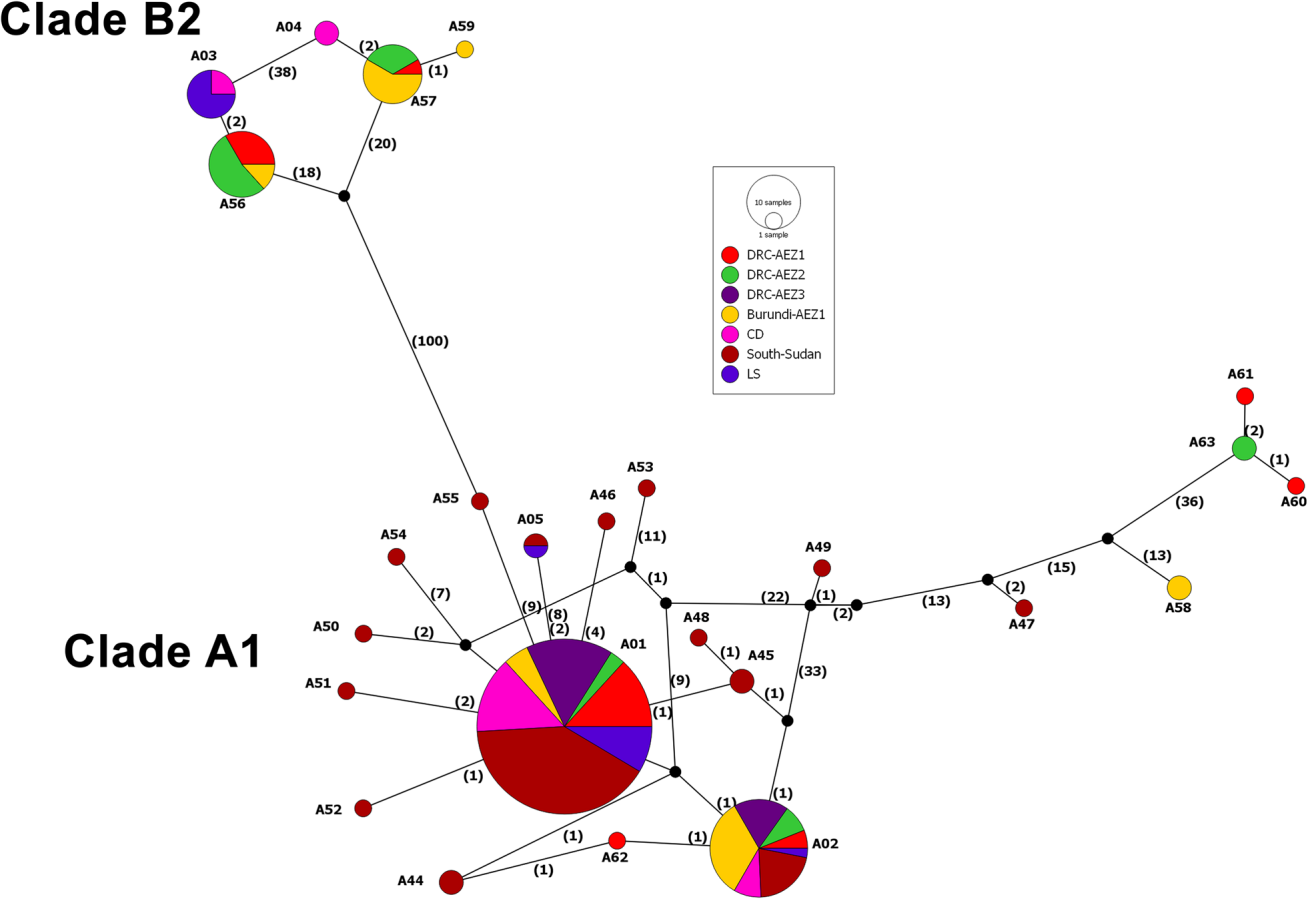
Median-joining network representing the phylogeographical distribution of *Tp2* alleles of *T. parva* from cattle in sub-Saharan Africa. Each circle represents a unique allele, with colours depicting the proportion of individuals from different populations sharing the allele. Black nodes represent hypothetical unsampled alleles (or median vectors). Numbers in brackets on connecting lines indicate mutational steps between alleles. The detailed *Tp2* alleles distribution and their corresponding populations/AEZs are presented in Additional file 10: Table S8. *Tp2* allele A01 corresponds to samples identical to Muguga and Serengeti-transformed strains and *Tp2* allele A02 represents samples identical to Kiambu-5 strain. CD, cattle-derived samples (from Kenya); LS, laboratory samples (ILRI) [36]

The median-joining (MJ) network performed on the 200 cattle-derived *Tp2* individual sequences described in Africa (collapsed into 25 representative alleles) recovered two major genetic groups that diverged at least by 100 nucleotide mutational steps (Fig. 6). The two groups fully corresponded to cattle-derived alleles clustered in clades A1 and B2 detected in the *Tp2* gene tree (Fig. 5) and contained ubiquitous alleles that were shared by two to more populations. The majority of low-frequency alleles occurred in South Sudan and were closely connected to the dominant allele (A01) which was present in all the seven populations. Interestingly, The MJ network showed an extensive admixture of cattle-derived parasite populations from diverse geographical locations with high number of mutational step connections.

The evolution of loci was compared with the evolution of *T. parva* samples using a concatenated phylogenetic analysis performed on 93 *Tp1* + *Tp2* individual sequences from cattle in the Great Lakes region. In total, 19 representative alleles were defined in the concatenated sequences. The NJ tree of the concatenated dataset provided similar topologies that fully agreed with that obtained by the *Tp2* phylogenetic analysis (Figs. 5, 6), resulting into two well-defined main clades of cattle-derived parasites (Additional file 12: Figure S4). Each of these clades was significantly divided into two sub-clades strongly supported by their bootstrap values. The major clade (clade A), which contained the three Muguga cocktail vaccine alleles, included 70 (75%) concatenated sequences corresponding to alleles found in sub-clade A1 of the *Tp2* NJ tree, while the minor clade (clade B) contained samples clustered in sub-clade B2 of the *Tp2* phylogenetic tree (Fig. 5).

### Population differentiation

The partition of genetic diversity in *Tp2* sequences was further analysed using analysis of molecular variance (AMOVA) based on allelic variants from the four AEZs of the Great Lakes region and those from South Sudan, Kenya (cattle-derived: CD; buffalo-derived: BD; and buffalo-associated: BA) and the laboratory *T. parva* stocks (LS). The AMOVA results further supported the findings obtained with phylogenetic analyses, showing that most of the variation (75% of the total variation) was found between individuals within populations, whereas a relatively small amount of the total diversity was significantly explained by interpopulation divergence (25%, *P *< 0.001). To examine the degree of gene flow and genetic differentiation levels among *T. parva* populations, Wright’s fixation index (*F*_*ST*_) values were computed for each pairwise comparison between *Tp2* sequences from different geographical origins, alongside buffalo-derived *T. parva* sequences obtained from GenBank. Overall, pairwise comparison (*F*_*ST*_) values between different geographical areas and/or populations ranged from -0.02 (between LS and DRC AEZ1) to the greatest genetic divergence of 0.69 (between DRC AEZ2 and South Sudan) (Table 4). The *F*_*ST*_ statistic revealed interesting findings. First, *T. parva* isolates from the highlands of DRC (DRC AEZ3) and those from South Sudan were not genetically different (*F*_*ST*_ = − 0.003), showing a high degree of similarity between alleles. These two populations contained the highest number of samples carrying the Muguga cocktail vaccine component alleles (alleles 1 and 2) (Additional file 10: Table S8). Secondly, *T. parva* parasite samples from lowlands (DRC AEZ1 and Burundi AEZ1) and midlands (DRC AEZ2) were genetically distant from those of highlands (DRC AEZ3) and the ones from South Sudan. Thirdly, the laboratory isolates (most ancient isolates from different sub-Saharan countries) were neither significantly divergent from those from lowlands and midlands of DRC and Burundi nor from Kenyan field isolates (CD).

**Table 4:** Pairwise estimates of genetic distance among nine *T. parva* populations using *F*_*ST*_ statistic for nucleotide sequences of *Tp2*

Country	Population	DRC AEZ1	DRC AEZ2	DRC AEZ3	Burundi AEZ1	BA	BD	CD	South Sudan	LS
DRC	DRC AEZ1	–	0.008	< 0.001	0.2	< 0.001	0.001	0.4	< 0.001	0.4
	DRC AEZ2	0.17	–	< 0.001	0.06	< 0.001	0.004	< 0.001	< 0.001	0.07
	DRC AEZ3	0.20	0.58	–	< 0.001	< 0.001	< 0.001	0.04	0.3	< 0.001
Burundi	Burundi AEZ1	0.02	0.08	0.3	–	< 0.001	0.008	0.1	< 0.001	0.3
Kenya	BA	0.17	0.19	0.39	0.15	–	0.3	< 0.001	< 0.001	< 0.001
	BD	0.17	0.17	0.45	0.14	0.002	–	< 0.001	< 0.001	0.009
	CD	− 0.01	0.28	0.13	0.06	0.22	0.23	–	0.002	0.1
South Sudan	South Sudan	0.29	0.69	− 0.003	0.41	0.52	0.59	0.19	–	< 0.001
Laboratory samples	LS	− 0.02	0.08	0.35	0.009	0.15	0.14	0.04	0.48	–

*Notes: F*_*ST*_ values below the diagonal and *P*-values above the diagonal; The genetic differentiation was considered as low (*F*_*ST*_ between 0–0.05), intermediate (*F*_*ST*_ between 0.05–0.15), great (*F*_*ST*_ between 0.15–0.25) and very great (*F*_*ST*_ < 0.25). The sample sizes (number of sequences) used in each population are shown in (Additional file 9: Table S7, Additional file 10: Table S8)

*Abbreviations*: AEZ, agro-ecological zones; BD, buffalo-derived; LS, laboratory samples; CD, cattle-derived

### Evolutionary population dynamics: evidence of immune selection or demographic processes?

The evolutionary dynamics of *T. parva* isolates from cattle in different geographical areas in sub-Saharan Africa were assessed by neutrality statistics and mismatch analyses to elucidate natural selection and demographic forces responsible for maintaining the observed polymorphism in the *Tp2* gene. We applied Tajima’s *D* and Fu and Liʼs D* and F* statistics to assess the mode and significance of any departure from neutral expectations for the entire sequence (Table 5) and using a sliding-window through the sequence (Fig. 3a). Overall, these analyses showed significant departure from neutral evolution expectations. These statistics, together with the multimodal mismatch pattern, confirmed the significant deviation from population expansion for the majority of studied populations. However, the evidence of population expansion signature was detected only in South Sudan population where the neutrality statistics were negative and significant (Table 5). For other populations, positive and significant values of Fu and Liʼs D* and F* statistics consistently observed in most areas suggested a significant pressure of balancing selection or diversifying selection that might be the reason of increased allelic frequency and nucleotide diversity, acting to maintain *Tp2* alleles at intermediate frequencies compared with expectations under neutrality (Table 2). In addition, the sliding-window plot showed that *Tp2* gene region of nucleotide positions 200–300 was the more diverse and subjected to positive values of Tajima’s *D* statistic (Fig. 3a, b). This region contains sequences for *Tp2* epitopes 4 and 5 and another part that does not contain defined epitopes, suggesting that the evolutionary pressure signature is randomly distributed within the gene.

**Table 5:** *Tp2*-based demographic structure and natural selection analyses of *T. parva* populations

Country	Population	Sample size	Tajima’s *D*	Fu and Liʼs D*	Fu and Liʼs F*
DRC	DRC AEZ1	25	1.2	0.97	0.94
	DRC AEZ2	20	2.5	1.8**	2.1**
	DRC AEZ3	23	1.1	0.84	1.1
Burundi	Burundi AEZ1	28	2.2	1.9**	2.1**
	*Tp2* Clade A	68	− 1.2	1.9**	0.6
	*Tp2* Clade B	28	3.6**	1.6**	2.6**
Overall (DRC and Burundi)		96	2.5	2.3**	2.3**
Kenya	CD	22	0.17	1.8**	1.4
South Sudan	South Sudan	65	− 2.5**	− 2.7*	− 3.1*
Laboratory samples	LS	17	2.7**	1.7**	2.3**

*P < 0.05, **P < 0.01

*Abbreviations*: AEZ, agro-ecological zone; LS, laboratory samples; CD, cattle-derived

## Discussion

The rationalisation and implementation of an effective ECF vaccine-based control require information of the circulating parasite antigenic variants in a region to assure vaccine efficacy (when vaccinated animals are exposed to wild parasites) and safety (cross-immunity is required if vaccine stock is transmitted by ticks to an immune cattle population). Previous genetic studies of *T. parva* schizont-infected cell lines and parasite field isolates from cattle and African buffalo in East Africa using schizont antigen genes revealed an extensive genetic and antigenic diversity in *T. parva* populations, which was much greater in buffalo than in cattle-derived parasites [24, 35–38]. In this study, we conducted a comprehensive analysis of *Tp1* and *Tp2* sequences to investigate the extent of diversity, the phylogenetic relationships and the evolutionary dynamics of *T. parva* samples obtained from cattle in four AEZs in the African Great Lakes region and determine how they relate to vaccine stocks and published sequences from various geographical areas of sub-Saharan Africa. We were particularly interested in understanding the role of agro-ecological conditions and anthropogenic movements of cattle in the genetic structuring and evolutionary dynamics of *T. parva*.

### *Theileria parva* populations are more variable in lowlands than highlands but ubiquitous alleles are identical to the Muguga vaccine components

The sequence analyses provided evidence of polymorphism at the nucleotide and amino acid levels and within the epitope-containing regions of the two genes in the *T. parva* population from the Great Lakes region. Genetic distance statistics showed particularly a higher level of similarity within *Tp1* sequences and an extensive diversity within *Tp2* sequences, supporting the evidence that the genetic diversity is greater in *Tp2* than in *Tp1* gene as previously reported [35, 36, 38]. Nevertheless, the major alleles and epitope variants identified in the two genes were identical to those found in the Muguga cocktail vaccine components. Besides, the genetic diversity results further showed that the parasite populations from highlands were less diverse compared to those from lowlands (DRC AEZ1 and Burundi AEZ1) and midlands (DRC AEZ2), which contained the majority of the genetic variation observed in the Great Lakes region. Interestingly, all the AEZs consistently shared the Muguga cocktail vaccine component alleles, that were the most ubiquitous in the region. The fact that sequences identical to the alleles in the Muguga cocktail were the most prevalent and broadly distributed may be associated with the reported unrestricted movement of cattle in the region [2–6]. Altogether, these findings indicate that the Muguga cocktail component alleles seem to be endemic and the most transmitted and circulating genotypes in the Great Lakes region, while their coexistence with other genetically distant and more diverse alleles in lowlands and midlands areas might be generating epidemics or unstable endemic situations. Furthermore, nucleotide sequence analysis of *T. parva* at the sub-Saharan African level revealed that cattle-derived *T. parva* populations circulating in the Great Lakes region, especially from lowlands and midlands of DRC and Burundi are more diverse in comparison with those reported in cattle from various ecological zones of sub-Saharan Africa [24, 36, 37]. These results further suggest that the level of allelic variation of *T. parva* may be significantly affected by the demographic processes such as broad geographical dispersal of the parasite populations through human population migration with their cattle, which consequently result in high connectivity between cattle populations in Africa [2, 3, 52].

### Limited population structure and geographic separation of *T. parva*

A comprehensive phylogenetic analysis of *T. parva Tp2* sequences from the Great Lakes region and those from other regions across sub-Saharan Africa strongly support the evidence that *T. parva* parasites circulating in cattle from the Great Lakes region are highly diverse, containing individuals similar to those found in cattle from most east African countries and newly described alleles [24, 36, 37]. The topologies derived from phylogenetic analysis deduced from the concatenated cattle-derived sequences (*Tp1* + *Tp2*) found in the Great Lakes region produced strong congruent results with *Tp2* analysis, suggesting that the two loci co-evolve with similar substitution patterns in cattle-derived *T. parva* samples. The NJ and MJ network algorithms clustered *T. parva* sequences into two main groups (clades). The Muguga reference sequence noticeably clustered together with the majority of sequences in the larger clade while the smaller clade contained alleles that are genetically the most distant from the Muguga reference alleles. However, *T. parva* genetic groups were not clearly separated by geographical sub-structuring, as there were no population-specific clade or sub-clade consistently associated with geographical origins. In addition, despite the large overall nucleotide diversity of *Tp2* sequences, the diversity within each clade was lower, strongly suggesting that the overall genetic variation was predominantly affected by the genetic divergence among samples belonging to different clades and poorly among samples from different AEZs. These patterns further reflect a limited geographical segregation of *T. parva* genotypes which seems to be explained by the occurrence of most dominant alleles (Muguga component stocks) in all geographical areas. The reduced population structure could be the evidence that balancing selection acting on the genes studied or gene flow through cattle immigration is maintaining similar ubiquitous alleles in *T. parva* populations from distinct geographical regions [52, 53]. This was further supported by the AMOVA, which indicate that the sequence variation was substantiality higher between individuals within populations rather than among populations.

The degree of gene flow and genetic differentiation among the populations was assessed by estimating *F*_*ST*_ statistics, which investigate the level of population subdivision. Although the phylogenetic analysis did not give a clear population structure or geographical grouping, there is evidence of statistically significant genetic differentiation between *T. parva* populations, mostly due to the presence of some unshared and more diverse alleles that are exclusive to parasite populations from particular AEZs. Overall, high genetic differentiation was observed between lowlands and highlands, supported by the strong evidence that all *T*. *parva* samples found in highlands were closely related to the Muguga cocktail vaccine stocks. The lowlands and midlands of DRC and Burundi had similar levels of genotypic distribution and variation. These areas are relatively close and may exchange more genotypes through the dispersal of the parasites during short-distance seasonal movement of cattle. In addition, cattle movements are very extensive in the Ruzizi valley (lowlands of Burundi and DRC) which is an important entry point for imported cattle from neighbour countries [3–5].

### Ecological conditions driving tick population dynamics are suggested to be affecting the biogeographical distribution of *T. parva* genotypes

The observed pattern of genotypic distribution suggests that ecological parameters driving the phenology and establishment ability of tick lineages seem to be further affecting the transmission dynamics of *T. parva* and consequently its genetic diversity and structure. In the African Great Lakes region, AEZs are mainly differentiated by temperature and rainfall (affected by altitude), which are crucial factors underlining the ecology and population dynamics of the tick vector [8, 9, 54–56]. Thus, these environmental factors might be involved in determining the population structure of ticks as well as the transmission pattern of specific genotypes of the pathogen in different ecological conditions. Our previous findings of the population structure of the tick vector *R. appendiculatus* allow a direct linking with population structure of the pathogen at the agro-ecological level [6]. We found that the diversity of *R. appendiculatus* had a strong altitudinal gradient, being lower in highlands and more extensive in lowlands. With this evidence, we hypothesise that the association between *T. parva* genotypes and biogeographical areas could be explained by the climate factors affecting tick vector capacity [54, 55, 57]. The extensive genetic diversity of *T. parva* observed in lowlands and midlands appears to be supported by the intensity of tick activity in cattle which could increase the transmission dynamics of the parasite and multiple reinfection and coinfection events [28, 58–60]. In addition, the lowlands areas are ecologically more suitable for the sympatric coexistence of two lineages of the tick vector with different diapause behaviour, which may allow the temporal persistence of ticks on cattle and permanent transmission of the parasite [6]. Therefore, the repeated and permanent acquisition and continued transmission of parasites may result in genetic recombination among *T. parva* genotypes during their sexual reproduction stage in the tick and generate new genotypes in the parasite population [20]. In contrast, the low level of genetic diversity observed in highlands could be a result of reduced tick burden that may consequently reduce the transmission intensity of *T. parva* [6, 58, 59]. The likely lower transmission intensity in highlands could restrict the effective population size of the parasite and reduce its diversity.

### Lack of evidence for recent host immune selective pressure but suggested demographic processes affecting the evolutionary structure of *T. parva*

*Theileria parva* population dynamics were inferred using neutrality statistics in order to understand the factors underlying the observed genetic variability in Africa. The results of these statistics suggested that balancing selection occurred in most populations except in South Sudan where *T. parva* parasites appear to have experienced a sudden demographic expansion [37]. However, although neutrality statistics provided positive values suggesting evidence for balancing selection, which might arise as a result of selective pressure of host immunity and might increase the frequency distribution of polymorphisms, it is worth noting that this pattern of departure from neutral evolution can also be caused by demographic processes such as immigration dynamics and population colonisation and admixture [61]. Previous studies provided evidence of positive selection pressure for amino acid changes acting on *Tp1* and *Tp2* genes, but there was no sufficient evidence of host immune-based selection [36]. In addition, it seems that the evolutionary pressure is not predominantly directed to known epitope regions but is randomly distributed across the entire region of the gene. Thus, the observed selection and polymorphism could have arisen either through immune selection acting on epitopes presented by the bovine MHC class I and recognised by CD8^+^ T cells or most likely from demographic processes of the parasites due to range expansion through cattle movements. Moreover, a recent comparison of the polymorphism in the T cell epitopes of geographically distant *T. parva* parasite populations from buffalo showed that both populations consistently shared a large proportion of epitope variants, suggesting that the majority of variability found in the two genes is more ancient rather than a result of recent immune-based substitutions [35]. This was further supported by the lack of genetic differentiation between the more diverse *T. parva* populations from DRC and Burundi and the ancient laboratory isolates from cattle in various geographical areas of Africa. It was also suggested that variation observed in cattle-derived parasites may represent the ancient diversity evolved in buffalo and that only a subset founder population have been established within cattle population [35, 36]. We can therefore suggest that the genetic distribution and variation of *T. parva* observed in the Great Lakes region are more affected by cattle translocation between populations (gene flow) and ecological traits regulating tick populations than the host immune pressure and other mechanisms such as selection, mutation and genetic drift (or bottlenecks).

### The use of the trivalent Muguga vaccine is not expected to introduce new *T. parva* antigenic variants

The findings of this study provided a broad picture of the genetic structure of *T. parva* in the African Great Lakes region as a baseline for future fine scale description of the parasite population and immunisation trials of ITM vaccine. However, the prevalence and the number of *T. parva* genotypes circulating in the region may be underestimated, as some of the strains have a shorter carrier state and low parasitaemia below the detection threshold of antigen markers in asymptomatic cattle sampled during a cross-sectional survey [13]. Longitudinal monitoring of infections could be suggested in order to understand the spatiotemporal dynamics of *T. parva* genotypes in the region and further molecular characterisation could be undertaken using multilocus markers [29, 62] and high-throughput sequencing approach [35, 63] or cloning parasites from individual animals to detect all possible diversity profiles. Of interest, the majority of *T. parva* samples analysed in this study have shown to carry alleles identical or nearly similar to Muguga cocktail vaccine strains, although an extensive diversity was observed in lowlands and midlands. The wide distribution of the vaccine alleles in the region may be used as reference point for vaccine trial composed with Muguga cocktail stocks to evaluate cross-immunity in field conditions using local strains as challenge without any risk of introducing new parasite variants. A particularly striking finding was that some alleles found in cattle from lowlands and midlands were close to alleles present in buffalo-derived parasites. These antigenic variants may break through immunity induced by the Muguga vaccine [15, 16]. Thus, it could be relevant to test an improved alternative vaccine in lowlands and midlands areas that include local parasite stocks to provide broad protection. In order to initiate a vaccination trial, the MHC class I diversity in cattle from the Great Lakes region could be assessed because of the differential immune responses between cattle of different MHC class I haplotypes [64].

## Conclusions

The present study sheds light on the strong genetic similarity among major *T. parva* genotypes circulating in the region and Muguga vaccine stocks. The high degree of variation observed within populations and the moderate agro-ecological sub-structuring suggested that *T. parva* genotypes evolving in cattle are circulating within and between African countries through short and long-distance cattle movement. The findings reported in this study also provide insight into factors affecting the population genetic structure and biogeographical distribution of *T. parva* in the African Great Lakes region. It appears that the local persistence and the geographical distribution of *T. parva* genotypes are mainly driven by ecological factors affecting tick vector population dynamics and competence. Furthermore, the widespread of major genotypes and the signature of selection are most probably related to extensive gene flow through cattle immigration and agro-ecological conditions determining the transmission intensity of *T. parva* rather than a recent mutational process of immune selective pressure. The observed patterns of genetic structure and diversity of *T. parva* indicate that the strong genotypic diversity found in the region would be generating ECF endemic instability in lowlands and midlands and an epidemic structure in highlands. However, the fact that ubiquitous alleles are genetically similar to those used in the Muguga vaccine, along with the high level of admixture, partially provides evidence for safe deployment of existing trivalent live vaccine for field trial without any risk of introducing new parasite variants in the Great Lakes region. The Muguga cocktail ITM vaccine trial could be implemented regardless of agro-ecological zone since animal movement plays an important role in the spread of major genotypes. Future efforts should be done to understand the vector-pathogen and host-pathogen genotype relationships in the transmission system and the spatiotemporal dynamics of *T. parva* genotypes.

## Supplementary information

**Additional file 1: Table S1.** Cattle blood sample distribution across agro-ecological zones.

**Additional file 2: Table S2.** Nucleotide and amino acid sequences of *Tp1* and *Tp2* antigen epitopes from *T. parva* Muguga reference sequence.

**Additional file 3: Table S3.** Characteristics of 119 *T. parva* samples obtained from cattle in different agro-ecological zones (AEZs) of The Democratic Republic of Congo and Burundi.

**Additional file 4: Figure S1.** Multiple sequence alignment of the 11 *Tp1* gene alleles obtained in this study.

**Additional file 5: Table S4.** Estimates of evolutionary divergence between gene alleles for *Tp1* and *Tp2*, using proportion nucleotide distance.

**Additional file 6: Table S5.** *Tp1* and *Tp2* genes alleles with their corresponding antigen variants.

**Additional file 7: Table S6.** Amino acid variants of *Tp1* and *Tp2* CD8^+^ T cell target epitopes of *T. parva* from DRC and Burundi.

**Additional file 8: Figure S2.** Multiple sequence alignment of the 10 *Tp2* gene alleles obtained in this study.

**Additional file 9: Table S7.** Distribution of *Tp1* gene alleles of *T. parva* from cattle and buffalo in the sub-Saharan region of Africa.

**Additional file 10: Table S8.** Distribution of *Tp2* gene alleles of *T. parva* from cattle and buffalo in the sub-Saharan region of Africa.

**Additional file 11: Figure S3.** Neighbor-joining tree showing phylogenetic relationships among 48 *Tp1* gene alleles described in Africa.

**Additional file 12: Figure S4.** Phylogenetic tree showing the relationships among concatenated *Tp1* and *Tp2* nucleotide sequences of 93 *T. parva* samples from cattle in DRC and Burundi.

## Data Availability

The data that support the findings of this study are included in the article and its additional files. Novel sequences of *T. parva* are available in the GenBank database under accession numbers MF449288-MF449294 and MF449295-MF449302 for *Tp1* and *Tp2* antigen genes, respectively.

## Abbreviations

AEZ: agro-ecological zone
AMOVA: analysis of molecular variance
BLAST: basic local alignment search tool
*D*: Tajima’s neutrality test
DNA: deoxyribonucleic acid
DRC: Democratic Republic of the Congo
ECF: East Coast fever
*Fs*: Fu’s neutrality tests
*F*_*ST*_: Wright’s fixation index
gDNA: genomic deoxyribonucleic acid
ITM: infection and treatment method
MHC: major histocompatibility complex
MJ: median-joining
*N*_*A*_: number of distinct alleles
NCBI: National Center for Biotechnology Information
NJ: neighbor-joining
nPCR: nested polymerase chain reaction
PCR: polymerase chain reaction
*S*: segregating sites
SD: standard deviation
SNPs: single-nucleotide polymorphisms
π: nucleotide diversity
