# Supplementary material for: Genetic and antigenic variation of the bovine tick-borne pathogen Theileria parva in the Great Lakes region of Central Africa
Source: Parasit Vectors. 2019 Dec 16;12:588. doi: 10.1186/s13071-019-3848-2 (PMC6915983; doi:10.1186/s13071-019-3848-2)
Supplement: Supplementary file 1 — Additional file 1: Table S1. Cattle blood sample distribution across agro-ecological zones. [file 13071_2019_3848_MOESM1_ESM.docx]

**Additional file 1: Table S1.** Cattle blood sample distribution across agro-ecological zones

| Country | Agro-ecological  zone (AEZ) | Blood sample (no. of cattle) | Positive samples to p104 |
| --- | --- | --- | --- |
| DRC | AEZ1 | 110 | 31 |
|  | AEZ2 | 114 | 28 |
|  | AEZ3 | 130 | 26 |
| Burundi | AEZ1 | 126 | 34 |
| Total |  | 480 | 119 |

DRC (AEZ1: Lowlands, AEZ2: Midlands, AEZ3: Highlands); Burundi (AEZ1: Lowlands)
