# Supplementary material for: Genetic and antigenic variation of the bovine tick-borne pathogen Theileria parva in the Great Lakes region of Central Africa
Source: Parasit Vectors. 2019 Dec 16;12:588. doi: 10.1186/s13071-019-3848-2 (PMC6915983; doi:10.1186/s13071-019-3848-2)
Supplement: Supplementary file 2 — Additional file 2: Table S2. Nucleotide and amino acid sequences of Tp1 and Tp2 antigen epitopes from T. parva Muguga reference sequence. [file 13071_2019_3848_MOESM2_ESM.docx]

Additional file 2: Table S2. Nucleotide and amino acid sequences of *Tp1* and *Tp2* antigen epitopes from *T. parva* Muguga reference sequence

| **Gene locus** | **Epitope positions with inner primers used in the present study** | | | **Published epitopes using outer primers (**GenBank JF451936 and JF451856 for Tp1 and Tp2, respectively) [34, 36] |
| --- | --- | --- | --- | --- |
|  | **Epitope** | **Amino acid sequence*** | **Nucleotide sequence*** |  |
| *Tp1* | Tp1_35-45_ | ^35^VGYPKVKEEML^45^ | ^104^GTAGGGTATCCAAAGGTTAAAGAAGAAATGCTA^136^ | Tp1_214-224_ |
| *Tp2* | Tp2_20-30_ | ^20^SHEELKKLGML^30^ | ^58^AGTCATGAAGAACTAAAAAAATTGGGAATGCTA^90^ | Tp2_27–37_ |
|  | Tp2_33-41_ | ^33^DGFDRDALF^41^ | ^97^GATGGTTTCGACAGGGATGCATTGTTC^123^ | Tp2_40–48_ |
|  | Tp2_42-52_ | ^42^KSSHGMGKVGK^52^ | ^124^AAATCATCACATGGTATGGGAAAGGTAGGAAAA^156^ | Tp2_49–59_ |
|  | Tp2_89-97_ | ^89^FAQSLVCVL^97^ | ^265^TTTGCACAAAGCCTAGTGTGCGTATTA^291^ | Tp2_96–104_ |
|  | Tp2_91-99_ | ^91^QSLVCVLMK^99^ | ^271^CAAAGCCTAGTGTGCGTATTAATGAAA^297^ | Tp2_98–106_ |
|  | Tp2_131-140_ | ^131^KTSIPNPCKW^140^ | ^391^AAAACAAGTATTCCAAATCCATGTAAATGG^420^ | Tp2_138–147_ |

*The numbers flanking epitope sequences represent their positions in the *Tp1* and *Tp2* antigen gene fragment

**References**

1. Graham SP, Pelle R, Yamage M, Mwangi DM, Honda Y, Mwakubambanya RS, et al. Characterization of the fine specificity of bovine CD8 T-cell responses to defined antigens from the protozoan parasite *Theileria parva*. Infect Immun. 2008;76(2):685-94.
2. Pelle R, Graham SP, Njahira MN, Osaso J, Saya RM, Odongo DO, et al. Two *Theileria parva* CD8 T cell antigen genes are more variable in buffalo than cattle parasites, but differ in pattern of sequence diversity. PLoS One. 2011;6(4):e19015.
