# Supplementary material for: Genetic and antigenic variation of the bovine tick-borne pathogen Theileria parva in the Great Lakes region of Central Africa
Source: Parasit Vectors. 2019 Dec 16;12:588. doi: 10.1186/s13071-019-3848-2 (PMC6915983; doi:10.1186/s13071-019-3848-2)
Supplement: Supplementary file 3 — Additional file 3: Table S3. Characteristics of 119 T. parva samples obtained from cattle in different agro-ecological zones (AEZs) of The Democratic Republic of Congo and Burundi. [file 13071_2019_3848_MOESM3_ESM.docx]

Additional file 3: Table S3. Characteristics of 119 *T. parva* samples obtained from cattle in different agro-ecological zones (AEZs) of The Democratic Republic of Congo and Burundi

| **N** | **Sample ID** | **Sample origin** | | | ***Tp1* locus** | | | | ***Tp2* locus** | | | | |
| --- | --- | --- | --- | --- | --- | --- | --- | --- | --- | --- | --- | --- | --- |
|  |  | **District** | **Country** | **AEZ^a^** | **Gene allele** | **Antigen variant** | **GenBank No. ^b^** | **Cluster** |  | **Gene allele** | **Antigen variant** | **GenBank No. ^b^** | **Cluster** |
| 1 | B01 | Rugombo | Burundi | 1 | 4 | 3 | JF451973 | 1 |  | 1 | 1 | JF451856 | 1A |
| 2 | B02 | Rugombo | Burundi | 1 | 1 | 1 | JF451936 | 1 |  | 58 | 55 | MF449297 | 1B |
| 3 | B03 | Rugombo | Burundi | 1 | 1 | 1 | JF451936 | 1 |  | 57 | 54 | MF449296 | 2A |
| 4 | B04 | Rugombo | Burundi | 1 | nd | nd | nd | nd |  | 58 | 55 | MF449297 | 1B |
| 5 | B05 | Rugombo | Burundi | 1 | 4 | 3 | JF451973 | 1 |  | 2 | 2 | JF451880 | 1A |
| 6 | B06 | Rugombo | Burundi | 1 | 37 | 31 | KJ566597 | 1 |  | 2 | 2 | JF451880 | 1A |
| 7 | B07 | Rugombo | Burundi | 1 | 37 | 31 | KJ566597 | 1 |  | 59 | 54 | MF449298 | 2A |
| 8 | B08 | Rugombo | Burundi | 1 | 1 | 1 | JF451936 | 1 |  | 2 | 2 | JF451880 | 1A |
| 9 | B09 | Rugombo | Burundi | 1 | 1 | 1 | JF451936 | 1 |  | 2 | 2 | JF451880 | 1A |
| 10 | B10 | Rugombo | Burundi | 1 | 1 | 1 | JF451936 | 1 |  | 1 | 1 | JF451856 | 1A |
| 11 | B11 | Rugombo | Burundi | 1 | 37 | 31 | KJ566597 | 1 |  | 1 | 1 | JF451856 | 1A |
| 12 | B12 | Rugombo | Burundi | 1 | 37 | 31 | KJ566597 | 1 |  | 57 | 54 | MF449296 | 2A |
| 13 | B13 | Rugombo | Burundi | 1 | 1 | 1 | JF451936 | 1 |  | 2 | 2 | JF451880 | 1A |
| 14 | B14 | Rugombo | Burundi | 1 | 1 | 1 | JF451936 | 1 |  | 2 | 2 | JF451880 | 1A |
| 15 | B15 | Rugombo | Burundi | 1 | 1 | 1 | JF451936 | 1 |  | 2 | 2 | JF451880 | 1A |
| 16 | B16 | Rugombo | Burundi | 1 | 4 | 3 | JF451973 | 1 |  | 56 | 53 | MF449295 | 2B |
| 17 | B17 | Rugombo | Burundi | 1 | 37 | 31 | KJ566597 | 1 |  | 57 | 54 | MF449296 | 2A |
| 18 | B18 | Rugombo | Burundi | 1 | 4 | 3 | JF451973 | 1 |  | 2 | 2 | JF451880 | 1A |
| 19 | B19 | Rugombo | Burundi | 1 | 1 | 1 | JF451936 | 1 |  | 2 | 2 | JF451880 | 1A |
| 20 | B20 | Gihanga | Burundi | 1 | 1 | 1 | JF451936 | 1 |  | 56 | 53 | MF449295 | 2B |
| 21 | B21 | Gihanga | Burundi | 1 | 1 | 1 | JF451936 | 1 |  | 1 | 1 | JF451856 | 1A |
| 22 | B22 | Gihanga | Burundi | 1 | 1 | 1 | JF451936 | 1 |  | 2 | 2 | JF451880 | 1A |
| 23 | B23 | Gihanga | Burundi | 1 | 1 | 1 | JF451936 | 1 |  | 2 | 2 | JF451880 | 1A |
| 24 | B24 | Gihanga | Burundi | 1 | 37 | 31 | KJ566597 | 1 |  | 57 | 54 | MF449296 | 2A |
| 25 | B25 | Gihanga | Burundi | 1 | 37 | 31 | KJ566597 | 1 |  | 57 | 54 | MF449296 | 2A |
| 26 | B26 | Gihanga | Burundi | 1 | 37 | 31 | KJ566597 | 1 |  | 57 | 54 | MF449296 | 2A |
| 27 | B27 | Gihanga | Burundi | 1 | 37 | 31 | KJ566597 | 1 |  | 57 | 54 | MF449296 | 2A |
| 28 | B28 | Gihanga | Burundi | 1 | 1 | 1 | JF451936 | 1 |  | 1 | 1 | JF451856 | 1A |
| 29 | B29 | Gihanga | Burundi | 1 | 1 | 1 | JF451936 | 1 |  | nd | nd | nd | nd |
| 30 | B30 | Gihanga | Burundi | 1 | 1 | 1 | JF451936 | 1 |  | nd | nd | nd | nd |
| 31 | B31 | Gihanga | Burundi | 1 | 1 | 1 | JF451936 | 1 |  | nd | nd | nd | nd |
| 32 | B32 | Gihanga | Burundi | 1 | 1 | 1 | JF451936 | 1 |  | nd | nd | nd | nd |
| 33 | B33 | Gihanga | Burundi | 1 | 37 | 31 | KJ566597 | 1 |  | nd | nd | nd | nd |
| 34 | B34 | Gihanga | Burundi | 1 | 37 | 31 | KJ566597 | 1 |  | nd | nd | nd | nd |
| 35 | K01 | Kabare | DRC | 3 | 1 | 1 | JF451936 | 1 |  | 1 | 1 | JF451856 | 1A |
| 36 | K02 | Kabare | DRC | 3 | 1 | 1 | JF451936 | 1 |  | 2 | 2 | JF451880 | 1A |
| 37 | K03 | Kabare | DRC | 3 | 1 | 1 | JF451936 | 1 |  | 2 | 2 | JF451880 | 1A |
| 38 | K04 | Kabare | DRC | 3 | 1 | 1 | JF451936 | 1 |  | 1 | 1 | JF451856 | 1A |
| 39 | K05 | Kabare | DRC | 3 | 1 | 1 | JF451936 | 1 |  | 1 | 1 | JF451856 | 1A |
| 40 | K06 | Kabare | DRC | 3 | 1 | 1 | JF451936 | 1 |  | 1 | 1 | JF451856 | 1A |
| 41 | K07 | Kabare | DRC | 3 | 1 | 1 | JF451936 | 1 |  | 1 | 1 | JF451856 | 1A |
| 42 | K08 | Kabare | DRC | 3 | 1 | 1 | JF451936 | 1 |  | 1 | 1 | JF451856 | 1A |
| 43 | K09 | Kabare | DRC | 3 | 1 | 1 | JF451936 | 1 |  | 2 | 2 | JF451880 | 1A |
| 44 | K10 | Kabare | DRC | 3 | 1 | 1 | JF451936 | 1 |  | 1 | 1 | JF451856 | 1A |
| 45 | K11 | Kabare | DRC | 3 | 1 | 1 | JF451936 | 1 |  | 2 | 2 | JF451880 | 1A |
| 46 | K12 | Kabare | DRC | 3 | 1 | 1 | JF451936 | 1 |  | 2 | 2 | JF451880 | 1A |
| 47 | K13 | Kabare | DRC | 3 | nd | nd | nd | nd |  | 1 | 1 | JF451856 | 1A |
| 48 | K14 | Kabare | DRC | 3 | 1 | 1 | JF451936 | 1 |  | 1 | 1 | JF451856 | 1A |
| 49 | K15 | Kabare | DRC | 3 | 1 | 1 | JF451936 | 1 |  | 1 | 1 | JF451856 | 1A |
| 50 | K16 | Kabare | DRC | 3 | 1 | 1 | JF451936 | 1 |  | 1 | 1 | JF451856 | 1A |
| 51 | K17 | Kabare | DRC | 3 | 1 | 1 | JF451936 | 1 |  | 1 | 1 | JF451856 | 1A |
| 52 | K18 | Kabare | DRC | 3 | 1 | 1 | JF451936 | 1 |  | 1 | 1 | JF451856 | 1A |
| 53 | K19 | Kabare | DRC | 3 | 1 | 1 | JF451936 | 1 |  | 2 | 2 | JF451880 | 1A |
| 54 | K20 | Kabare | DRC | 3 | 1 | 1 | JF451936 | 1 |  | 1 | 1 | JF451856 | 1A |
| 55 | K21 | Kabare | DRC | 3 | 1 | 1 | JF451936 | 1 |  | 1 | 1 | JF451856 | 1A |
| 56 | K22 | Kabare | DRC | 3 | 1 | 1 | JF451936 | 1 |  | 1 | 1 | JF451856 | 1A |
| 57 | K23 | Kabare | DRC | 3 | 1 | 1 | JF451936 | 1 |  | 1 | 1 | JF451856 | 1A |
| 58 | K24 | Kabare | DRC | 3 | 1 | 1 | JF451936 | 1 |  | nd | nd | nd | nd |
| 59 | K25 | Kabare | DRC | 3 | 1 | 1 | JF451936 | 1 |  | nd | nd | nd | nd |
| 60 | K26 | Kabare | DRC | 3 | 1 | 1 | JF451936 | 1 |  | nd | nd | nd | nd |
| 61 | U01 | Uvira | DRC | 1 | 1 | 1 | JF451936 | 1 |  | 1 | 1 | JF451856 | 1A |
| 62 | U02 | Uvira | DRC | 1 | 1 | 1 | JF451936 | 1 |  | 1 | 1 | JF451856 | 1A |
| 63 | U03 | Uvira | DRC | 1 | 1 | 1 | JF451936 | 1 |  | 56 | 53 | MF449295 | 2B |
| 64 | U04 | Uvira | DRC | 1 | 1 | 1 | JF451936 | 1 |  | 1 | 1 | JF451856 | 1A |
| 65 | U05 | Uvira | DRC | 1 | 1 | 1 | JF451936 | 1 |  | 1 | 1 | JF451856 | 1A |
| 66 | U06 | Uvira | DRC | 1 | 1 | 1 | JF451936 | 1 |  | 1 | 1 | JF451856 | 1A |
| 67 | U07 | Uvira | DRC | 1 | 37 | 31 | KJ566597 | 1 |  | 1 | 1 | JF451856 | 1A |
| 68 | U08 | Uvira | DRC | 1 | 37 | 31 | KJ566597 | 1 |  | 1 | 1 | JF451856 | 1A |
| 69 | U09 | Uvira | DRC | 1 | 1 | 1 | JF451936 | 1 |  | 1 | 1 | JF451856 | 1A |
| 70 | U10 | Uvira | DRC | 1 | 1 | 1 | JF451936 | 1 |  | 1 | 1 | JF451856 | 1A |
| 71 | U11 | Uvira | DRC | 1 | 1 | 1 | JF451936 | 1 |  | 1 | 1 | JF451856 | 1A |
| 72 | U12 | Uvira | DRC | 1 | 4 | 3 | JF451973 | 1 |  | 56 | 53 | MF449295 | 2B |
| 73 | U13 | Uvira | DRC | 1 | 4 | 3 | JF451973 | 1 |  | 56 | 53 | MF449295 | 2B |
| 74 | U14 | Uvira | DRC | 1 | 4 | 3 | JF451973 | 1 |  | 2 | 2 | JF451880 | 1A |
| 75 | U15 | Uvira | DRC | 1 | 1 | 1 | JF451936 | 1 |  | 1 | 1 | JF451856 | 1A |
| 76 | U16 | Uvira | DRC | 1 | 1 | 1 | JF451936 | 1 |  | 1 | 1 | JF451856 | 1A |
| 77 | U17 | Uvira | DRC | 1 | 1 | 1 | JF451936 | 1 |  | 1 | 1 | JF451856 | 1A |
| 78 | U18 | Uvira | DRC | 1 | 37 | 31 | KJ566597 | 1 |  | 57 | 54 | MF449296 | 2A |
| 79 | U19 | Uvira | DRC | 1 | 1 | 1 | JF451936 | 1 |  | 2 | 2 | JF451880 | 1A |
| 80 | U20 | Uvira | DRC | 1 | 4 | 3 | JF451973 | 1 |  | 62 | 58 | MF449301 | 1A |
| 81 | U21 | Uvira | DRC | 1 | 1 | 1 | JF451936 | 1 |  | 1 | 1 | JF451856 | 1A |
| 82 | U22 | Uvira | DRC | 1 | 4 | 3 | JF451973 | 1 |  | 56 | 53 | MF449295 | 2B |
| 83 | U23 | Uvira | DRC | 1 | 37 | 31 | KJ566597 | 1 |  | 56 | 53 | MF449295 | 2B |
| 84 | U24 | Uvira | DRC | 1 | 45 | 32 | MF449290 | 2 |  | 60 | 56 | MF449299 | 1B |
| 85 | U25 | Uvira | DRC | 1 | 45 | 32 | MF449290 | 2 |  | 61 | 57 | MF449300 | 1B |
| 86 | U26 | Uvira | DRC | 1 | 43 | 1 | MF449288 | 1 |  | nd | nd | nd | nd |
| 87 | U27 | Uvira | DRC | 1 | 1 | 1 | JF451936 | 1 |  | nd | nd | nd | nd |
| 88 | U28 | Uvira | DRC | 1 | 1 | 1 | JF451936 | 1 |  | nd | nd | nd | nd |
| 89 | U29 | Uvira | DRC | 1 | 1 | 1 | JF451936 | 1 |  | nd | nd | nd | nd |
| 90 | U30 | Uvira | DRC | 1 | 44 | 3 | MF449289 | 1 |  | nd | nd | nd | nd |
| 91 | U31 | Uvira | DRC | 1 | 46 | 33 | MF449291 | 1 |  | nd | nd | nd | nd |
| 92 | W01 | Walungu | DRC | 2 | 1 | 1 | JF451936 | 1 |  | 1 | 1 | JF451856 | 1A |
| 93 | W02 | Walungu | DRC | 2 | 1 | 1 | JF451936 | 1 |  | 56 | 53 | MF449295 | 2B |
| 94 | W03 | Walungu | DRC | 2 | 39 | 1 | KJ566599 | 1 |  | 1 | 1 | JF451856 | 1A |
| 95 | W04 | Walungu | DRC | 2 | 1 | 1 | JF451936 | 1 |  | 2 | 2 | JF451880 | 1A |
| 96 | W05 | Walungu | DRC | 2 | 4 | 3 | JF451973 | 1 |  | 56 | 53 | MF449295 | 2B |
| 97 | W06 | Walungu | DRC | 2 | 37 | 31 | KJ566597 | 1 |  | 56 | 53 | MF449295 | 2B |
| 98 | W07 | Walungu | DRC | 2 | 37 | 31 | KJ566597 | 1 |  | 56 | 53 | MF449295 | 2B |
| 99 | W08 | Walungu | DRC | 2 | 1 | 1 | JF451936 | 1 |  | 56 | 53 | MF449295 | 2B |
| 100 | W09 | Walungu | DRC | 2 | 1 | 1 | JF451936 | 1 |  | 56 | 53 | MF449295 | 2B |
| 101 | W10 | Walungu | DRC | 2 | 1 | 1 | JF451936 | 1 |  | 56 | 53 | MF449295 | 2B |
| 102 | W11 | Walungu | DRC | 2 | 37 | 31 | KJ566597 | 1 |  | 56 | 53 | MF449295 | 2B |
| 103 | W12 | Walungu | DRC | 2 | 1 | 1 | JF451936 | 1 |  | 2 | 2 | JF451880 | 1A |
| 104 | W13 | Walungu | DRC | 2 | nd | nd | nd | nd |  | 1 | 1 | JF451856 | 1A |
| 105 | W14 | Walungu | DRC | 2 | 45 | 32 | MF449290 | 2 |  | 63 | 59 | MF449302 | 1B |
| 106 | W15 | Walungu | DRC | 2 | 45 | 32 | MF449290 | 2 |  | 63 | 59 | MF449302 | 1B |
| 107 | W16 | Walungu | DRC | 2 | 1 | 1 | JF451936 | 1 |  | 57 | 54 | MF449296 | 2A |
| 108 | W17 | Walungu | DRC | 2 | 1 | 1 | JF451936 | 1 |  | 57 | 54 | MF449296 | 2A |
| 109 | W18 | Walungu | DRC | 2 | 1 | 1 | JF451936 | 1 |  | 57 | 54 | MF449296 | 2A |
| 110 | W19 | Walungu | DRC | 2 | 1 | 1 | JF451936 | 1 |  | 2 | 2 | JF451880 | 1A |
| 111 | W20 | Walungu | DRC | 2 | 39 | 1 | KJ566599 | 1 |  | 57 | 54 | MF449296 | 2A |
| 112 | W21 | Walungu | DRC | 2 | 48 | 34 | MF449293 | 1 |  | nd | nd | nd | nd |
| 113 | W22 | Walungu | DRC | 2 | 47 | 1 | MF449292 | 1 |  | nd | nd | nd | nd |
| 114 | W23 | Walungu | DRC | 2 | 1 | 1 | JF451936 | 1 |  | nd | nd | nd | nd |
| 115 | W24 | Walungu | DRC | 2 | 1 | 1 | JF451936 | 1 |  | nd | nd | nd | nd |
| 116 | W25 | Walungu | DRC | 2 | 1 | 1 | JF451936 | 1 |  | nd | nd | nd | nd |
| 117 | W26 | Walungu | DRC | 2 | 1 | 1 | JF451936 | 1 |  | nd | nd | nd | nd |
| 118 | W27 | Walungu | DRC | 2 | 1 | 1 | JF451936 | 1 |  | nd | nd | nd | nd |
| 119 | W28 | Walungu | DRC | 2 | 49 | 33 | MF449294 | 1 |  | nd | nd | nd | nd |

*Notes*: Ninety-six and 116 reliable sequences were obtained for *Tp2* and *Tp1* gene loci respectively; Twenty-three samples failed to be amplified by *Tp2* gene and three samples failed for *Tp1* gene

^a^AEZ-1, lowlands valleys of DRC (Ruzizi valley) and Burundi (Imbo valley); AEZ-2, midlands of DRC in the Walungu district; AEZ-3, highlands of DRC in the Kabare district

^b^ GenBank accession numbers with JF and KJ prefixes were previously described in Kenya [36] and South Sudan [37], while GenBank accession numbers for newly described alleles are represented by MF prefix.

*Abbreviations*: AEZ, agro-ecological zones; nd, not determined.

**References**

1. Pelle R, Graham SP, Njahira MN, Osaso J, Saya RM, Odongo DO, et al. Two *Theileria parva* CD8 T cell antigen genes are more variable in buffalo than cattle parasites, but differ in pattern of sequence diversity. PLoS One. 2011;6(4):e19015.
2. Salih DA, Pelle R, Mwacharo JM, Njahira MN, Marcellino WL, Kiara H, et al. Genes encoding two *Theileria parva* antigens recognized by CD8+ T-cells exhibit sequence diversity in South Sudanese cattle populations but the majority of alleles are similar to the Muguga component of the live vaccine cocktail. PLoS One. 2017;12(2):e0171426.
