# Supplementary material for: Genetic and antigenic variation of the bovine tick-borne pathogen Theileria parva in the Great Lakes region of Central Africa
Source: Parasit Vectors. 2019 Dec 16;12:588. doi: 10.1186/s13071-019-3848-2 (PMC6915983; doi:10.1186/s13071-019-3848-2)
Supplement: Supplementary file 4 — Additional file 4: Figure S1. Multiple sequence alignment of the 11 Tp1 gene alleles obtained in this study. [file 13071_2019_3848_MOESM4_ESM.docx]

Additional file 4: Figure S1. Multiple sequence alignment of the 11 *Tp1* gene alleles obtained in this study. The flanked primer regions are shaded and boxed. The CD8+ T cell target epitope coding region and indel insertion are bolded and boxed. *Tp1* allele1 corresponds to samples identical with the three vaccine strains (Muguga, Serengeti-transformed and Kiambu-5).
