# Supplementary material for: Genetic and antigenic variation of the bovine tick-borne pathogen Theileria parva in the Great Lakes region of Central Africa
Source: Parasit Vectors. 2019 Dec 16;12:588. doi: 10.1186/s13071-019-3848-2 (PMC6915983; doi:10.1186/s13071-019-3848-2)
Supplement: Supplementary file 5 — Additional file 5: Table S4. Estimates of evolutionary divergence between gene alleles for Tp1 and Tp2, using proportion nucleotide distance. [file 13071_2019_3848_MOESM5_ESM.docx]

Additional file 5: Table S4. Estimates of evolutionary divergence between gene alleles for *Tp1* and *Tp2*, using proportion nucleotide distance

| ***Tp1*** | Allele-1 | Allele-4 | Allele-37 | Allele-39 | Allele-43 | Allele-44 | Allele-45 | Allele-46 | Allele-47 | Allele-48 | Allele-49 |
| --- | --- | --- | --- | --- | --- | --- | --- | --- | --- | --- | --- |
| Allele-1 |  |  |  |  |  |  |  |  |  |  |  |
| Allele-4 | 0.01 |  |  |  |  |  |  |  |  |  |  |
| Allele-37 | 0.007 | 0.002 |  |  |  |  |  |  |  |  |  |
| Allele-39 | 0.002 | 0.007 | 0.005 |  |  |  |  |  |  |  |  |
| Allele-43 | 0.002 | 0.012 | 0.010 | 0.005 |  |  |  |  |  |  |  |
| Allele-44 | 0.012 | 0.002 | 0.005 | 0.010 | 0.015 |  |  |  |  |  |  |
| Allele-45 | 0.017 | 0.025 | 0.022 | 0.017 | 0.020 | 0.027 |  |  |  |  |  |
| Allele-46 | 0.005 | 0.005 | 0.002 | 0.002 | 0.007 | 0.007 | 0.020 |  |  |  |  |
| Allele-47 | 0.002 | 0.012 | 0.010 | 0.005 | 0.005 | 0.015 | 0.020 | 0.007 |  |  |  |
| Allele-48 | 0.015 | 0.005 | 0.007 | 0.012 | 0.017 | 0.007 | 0.025 | 0.010 | 0.017 |  |  |
| Allele-49 | 0.002 | 0.007 | 0.005 | 0.005 | 0.005 | 0.010 | 0.020 | 0.002 | 0.005 | 0.012 |  |

| ***Tp2*** | Allele-1 | Allele-2 | Allele-56 | Allele-57 | Allele-58 | Allele-59 | Allele-60 | Allele-61 | Allele-62 | Allele-63 |
| --- | --- | --- | --- | --- | --- | --- | --- | --- | --- | --- |
| Allele-1 |  |  |  |  |  |  |  |  |  |  |
| Allele-2 | 0.004 |  |  |  |  |  |  |  |  |  |
| Allele-56 | 0.25 | 0.25 |  |  |  |  |  |  |  |  |
| Allele-57 | 0.26 | 0.25 | 0.075 |  |  |  |  |  |  |  |
| Allele-58 | 0.15 | 0.15 | 0.26 | 0.28 |  |  |  |  |  |  |
| Allele-59 | 0.26 | 0.25 | 0.077 | 0.002 | 0.28 |  |  |  |  |  |
| Allele-60 | 0.14 | 0.14 | 0.24 | 0.26 | 0.10 | 0.27 |  |  |  |  |
| Allele-61 | 0.14 | 0.14 | 0.25 | 0.27 | 0.10 | 0.27 | 0.006 |  |  |  |
| Allele-62 | 0.006 | 0.002 | 0.25 | 0.25 | 0.15 | 0.25 | 0.14 | 0.14 |  |  |
| Allele-63 | 0.14 | 0.14 | 0.24 | 0.26 | 0.10 | 0.27 | 0.002 | 0.004 | 0.14 |  |

*Notes*: Evolutionary divergence between genes alleles was estimated using proportion nucleotide distance in MEGA.

*Tp1* allele-1 corresponds to isolates identical to the three vaccine strains (Muguga, Serengeti-transformed and Kiambu-5).
*Tp2* allele-1 corresponds to isolates identical to Muguga and Serengeti-transformed strains and *Tp2* allele-2 represents samples identical to Kiambu-5 strain.
