# Supplementary material for: Genetic and antigenic variation of the bovine tick-borne pathogen Theileria parva in the Great Lakes region of Central Africa
Source: Parasit Vectors. 2019 Dec 16;12:588. doi: 10.1186/s13071-019-3848-2 (PMC6915983; doi:10.1186/s13071-019-3848-2)
Supplement: Supplementary file 6 — Additional file 6: Table S5. Tp1 and Tp2 genes alleles with their corresponding antigen variants. [file 13071_2019_3848_MOESM6_ESM.docx]

Additional file 6: Table S5. *Tp1* and *Tp2* genes alleles with their corresponding antigen variants

| **Marker** | **Gene alleles** | **Antigen variants** | **Number of *T. parva* samples** | **Frequency (%)** |
| --- | --- | --- | --- | --- |
| *Tp1* | Allele-1 | Var-1 | 76 | 65.5 |
|  | Allele-4 | Var-3 | 10 | 8.6 |
|  | Allele-37 | Var-31 | 18 | 15.5 |
|  | Allele-39 | Var-1 | 2 | 1.7 |
|  | Allele-43 | Var-1 | 1 | 0.9 |
|  | Allele-44 | Var-3 | 1 | 0.9 |
|  | Allele-45 | Var-32 | 4 | 3.4 |
|  | Allele-46 | Var-33 | 1 | 0.9 |
|  | Allele-47 | Var-1 | 1 | 0.9 |
|  | Allele-48 | Var-34 | 1 | 0.9 |
|  | Allele-49 | Var-33 | 1 | 0.9 |
|  | Total no. of *Tp1* sequences | | 116 | 100.0 |
| *Tp2* | Allele-1 | Var-1 | 39 | 40.6 |
|  | Allele-2 | Var-2 | 22 | 22.9 |
|  | Allele-56 | Var-53 | 15 | 15.6 |
|  | Allele-57 | Var-54 | 12 | 12.5 |
|  | Allele-58 | Var-55 | 2 | 2.1 |
|  | Allele-59 | Var-54 | 1 | 1.0 |
|  | Allele-60 | Var-56 | 1 | 1.0 |
|  | Allele-61 | Var-57 | 1 | 1.0 |
|  | Allele-62 | Var-58 | 1 | 1.0 |
|  | Allele-63 | Var-59 | 2 | 2.1 |
|  | Total no. of *Tp2* sequences | | 96 | 100.0 |
