# Supplementary material for: Genetic and antigenic variation of the bovine tick-borne pathogen Theileria parva in the Great Lakes region of Central Africa
Source: Parasit Vectors. 2019 Dec 16;12:588. doi: 10.1186/s13071-019-3848-2 (PMC6915983; doi:10.1186/s13071-019-3848-2)
Supplement: Supplementary file 7 — Additional file 7: Table S6. Amino acid variants of Tp1 and Tp2 CD8+ T cell target epitopes of T. parva from DRC and Burundi. [file 13071_2019_3848_MOESM7_ESM.docx]

Additional file 7: Table S6. Amino acid variants of *Tp1* and *Tp2* CD8^+^ T cell target epitopes of *T. parva* from DRC and Burundi

| **Epitope variants** | **DRC AEZ1 (25)** | **DRC AEZ2 (20)** | **DRC AEZ3 (23)** | **Burundi AEZ1 (28)** | **Overall (96)** |
| --- | --- | --- | --- | --- | --- |
| **Tp2_20-30_** |  |  |  |  |  |
| **SHEELKKLGML** | 14 | 3 | 17 | 5 | 39 |
| **SDEELNKLGML** | 2 | 3 | 6 | 11 | 22 |
| SDDELDTLGML | 5 | 8 | 0 | 2 | 15 |
| SDNELDTLGLL | 1 | 4 | 0 | 8 | 13 |
| TEEELRKLGMV | 2 | 2 | 0 | 0 | 4 |
| TEEELKKMGMV | 0 | 0 | 0 | 2 | 2 |
| SDEELNILGML | 1 | 0 | 0 | 0 | 1 |
| **Tp2_33-41_** |  |  |  |  |  |
| **DGFDRDALF** | 17 | 5 | 23 | 16 | 61 |
| PDLDKNRLF | 6 | 13 | 0 | 10 | 29 |
| SNFDRESLF | 2 | 2 | 0 | 0 | 4 |
| EGFDKEKLF | 0 | 0 | 0 | 2 | 2 |
| **Tp2_42-52_** |  |  |  |  |  |
| **KSSHGMGKVGK** | 17 | 6 | 23 | 16 | 62 |
| LTSHGMGRIGR | 5 | 8 | 0 | 2 | 15 |
| LTSHGMGKIGR | 1 | 4 | 0 | 8 | 13 |
| KSSHGMGKVGR | 2 | 2 | 0 | 0 | 4 |
| KSSKSMGIVGR | 0 | 0 | 0 | 2 | 2 |
| **Tp2_89-97_** |  |  |  |  |  |
| **FAQSLVCVL** | 17 | 6 | 23 | 16 | 62 |
| FAASIKCVA | 5 | 8 | 0 | 2 | 15 |
| LAASIKCVS | 1 | 4 | 0 | 8 | 13 |
| FAQSILCVI | 2 | 2 | 0 | 0 | 4 |
| FVQSIMCVI | 0 | 0 | 0 | 2 | 2 |
| **Tp2_91-99_** |  |  |  |  |  |
| **QSLVCVLMK** | 17 | 6 | 23 | 16 | 62 |
| ASIKCVAQY | 5 | 8 | 0 | 2 | 15 |
| ASIKCVSHH | 1 | 4 | 0 | 8 | 13 |
| QSILCVIKN | 2 | 2 | 0 | 0 | 4 |
| QSIMCVINK | 0 | 0 | 0 | 2 | 2 |
| **Tp2_131-140_** |  |  |  |  |  |
| **KTSIPNPCKW** | 17 | 6 | 23 | 16 | 62 |
| KPSVPNPCDW | 6 | 12 | 0 | 10 | 28 |
| ASDIPNPCKW | 2 | 2 | 0 | 0 | 4 |
| VNDIPNPCKW | 0 | 0 | 0 | 2 | 2 |
| **Tp1_35-45_** | **31** | **27** | **25** | **33** | **116** |
| **VGYPKVKEEML** | 20 | 21 | 25 | 18 | 84 |
| VGYPKVKEEII | 10 | 5 | 0 | 15 | 30 |
| VGYPKVKEEMI | 1 | 1 | 0 | 0 | 2 |

*Notes*: Numbers in bracket are sample size in each AEZ; Epitope sequences in bold are present in the reference sequences of *T. parva* stocks component of the live vaccine.
