# Supplementary material for: Genetic and antigenic variation of the bovine tick-borne pathogen Theileria parva in the Great Lakes region of Central Africa
Source: Parasit Vectors. 2019 Dec 16;12:588. doi: 10.1186/s13071-019-3848-2 (PMC6915983; doi:10.1186/s13071-019-3848-2)
Supplement: Supplementary file 8 — Additional file 8: Figure S2. Multiple sequence alignment of the 10 Tp2 gene alleles obtained in this study. [file 13071_2019_3848_MOESM8_ESM.docx]

Additional file 8: Figure S2. Multiple sequence alignment of the 10 *Tp2* gene alleles obtained in this study. The flanked primer regions are shaded and boxed. The CD8^+^ T cell target epitope coding regions (1-6) are bolded and boxed. *Tp2* allele-1 corresponds to *T. parva* samples identical to Muguga and Serengeti-transformed and *Tp2* allele-2 represents samples similar to Kiambu-5.
