# Supplementary material for: Genetic and antigenic variation of the bovine tick-borne pathogen Theileria parva in the Great Lakes region of Central Africa
Source: Parasit Vectors. 2019 Dec 16;12:588. doi: 10.1186/s13071-019-3848-2 (PMC6915983; doi:10.1186/s13071-019-3848-2)
Supplement: Supplementary file 9 — Additional file 9: Table S7. Distribution of Tp1 gene alleles of T. parva from cattle and buffalo in the sub-Saharan region of Africa. [file 13071_2019_3848_MOESM9_ESM.docx]

Additional file 9: Table S7. Distribution of *Tp1* gene alleles of *T. parva* from cattle and buffalo in the sub-Saharan region of Africa

| ***Tp1* alleles** | **The Great Lakes region** | | | | **Kenya** | | | **South Sudan** | **Laboratory samples** | **Total** |
| --- | --- | --- | --- | --- | --- | --- | --- | --- | --- | --- |
|  | DRC AEZ1 | DRC AEZ2 | DRC AEZ3 | Burundi AEZ1 | BA | BD | CD |  |  |  |
| A01 | 17 | 16 | 25 | 18 | 1 | 5 | 12 | 53 | 6 | **153** |
| A02 | 0 | 0 | 0 | 0 | 0 | 0 | 3 | 0 | 8 | **11** |
| A03 | 0 | 0 | 0 | 0 | 0 | 0 | 1 | 0 | 1 | **2** |
| A04 | 5 | 1 | 0 | 4 | 0 | 0 | 2 | 14 | 0 | **26** |
| A05 | 0 | 0 | 0 | 0 | 0 | 0 | 0 | 0 | 1 | **1** |
| A06 | 0 | 0 | 0 | 0 | 0 | 0 | 1 | 0 | 0 | **1** |
| A07 | 0 | 0 | 0 | 0 | 0 | 0 | 1 | 0 | 0 | **1** |
| A08 | 0 | 0 | 0 | 0 | 0 | 0 | 1 | 0 | 0 | **1** |
| A09 | 0 | 0 | 0 | 0 | 0 | 0 | 1 | 0 | 0 | **1** |
| A10 | 0 | 0 | 0 | 0 | 0 | 0 | 1 | 0 | 0 | **1** |
| A11 | 0 | 0 | 0 | 0 | 0 | 0 | 1 | 0 | 0 | **1** |
| A12 | 0 | 0 | 0 | 0 | 0 | 0 | 1 | 0 | 0 | **1** |
| A13 & 35 | 0 | 0 | 0 | 0 | 8 | 0 | 0 | 0 | 0 | **8** |
| A14 | 0 | 0 | 0 | 0 | 1 | 2 | 0 | 0 | 0 | **3** |
| A15 | 0 | 0 | 0 | 0 | 2 | 0 | 0 | 0 | 0 | **2** |
| A16 | 0 | 0 | 0 | 0 | 0 | 1 | 0 | 0 | 0 | **1** |
| A17 | 0 | 0 | 0 | 0 | 0 | 1 | 0 | 0 | 0 | **1** |
| A18 | 0 | 0 | 0 | 0 | 0 | 1 | 0 | 0 | 0 | **1** |
| A19 | 0 | 0 | 0 | 0 | 0 | 1 | 0 | 0 | 0 | **1** |
| A20 | 0 | 0 | 0 | 0 | 0 | 1 | 0 | 0 | 0 | **1** |
| A21 | 0 | 0 | 0 | 0 | 0 | 1 | 0 | 0 | 0 | **1** |
| A22 | 0 | 0 | 0 | 0 | 0 | 1 | 0 | 0 | 0 | **1** |
| A23 | 0 | 0 | 0 | 0 | 0 | 1 | 0 | 0 | 0 | **1** |
| A24 | 0 | 0 | 0 | 0 | 0 | 1 | 0 | 0 | 0 | **1** |
| A25 | 0 | 0 | 0 | 0 | 1 | 0 | 0 | 0 | 0 | **1** |
| A26 | 0 | 0 | 0 | 0 | 1 | 0 | 0 | 0 | 0 | **1** |
| A27 | 0 | 0 | 0 | 0 | 1 | 0 | 0 | 0 | 0 | **1** |
| A28 | 0 | 0 | 0 | 0 | 1 | 0 | 0 | 0 | 0 | **1** |
| A29 | 0 | 0 | 0 | 0 | 1 | 0 | 0 | 0 | 0 | **1** |
| A30 | 0 | 0 | 0 | 0 | 1 | 0 | 0 | 0 | 0 | **1** |
| A31 | 0 | 0 | 0 | 0 | 1 | 0 | 0 | 0 | 0 | **1** |
| A32 | 0 | 0 | 0 | 0 | 1 | 0 | 0 | 0 | 0 | **1** |
| A33 | 0 | 0 | 0 | 0 | 1 | 0 | 0 | 0 | 0 | **1** |
| A34 | 0 | 0 | 0 | 0 | 1 | 0 | 0 | 0 | 0 | **1** |
| A36 | 0 | 0 | 0 | 0 | 0 | 0 | 0 | 4 | 0 | **4** |
| A37 | 4 | 3 | 0 | 11 | 0 | 0 | 0 | 3 | 0 | **21** |
| A38 | 0 | 0 | 0 | 0 | 0 | 0 | 0 | 1 | 0 | **1** |
| A39 | 0 | 2 | 0 | 0 | 0 | 0 | 0 | 1 | 0 | **3** |
| A40 | 0 | 0 | 0 | 0 | 0 | 0 | 0 | 1 | 0 | **1** |
| A41 | 0 | 0 | 0 | 0 | 0 | 0 | 0 | 1 | 0 | **1** |
| A42 | 0 | 0 | 0 | 0 | 0 | 0 | 0 | 1 | 0 | **1** |
| A43 | 1 | 0 | 0 | 0 | 0 | 0 | 0 | 0 | 0 | **1** |
| A44 | 1 | 0 | 0 | 0 | 0 | 0 | 0 | 0 | 0 | **1** |
| A45 | 2 | 2 | 0 | 0 | 0 | 0 | 0 | 0 | 0 | **4** |
| A46 | 1 | 0 | 0 | 0 | 0 | 0 | 0 | 0 | 0 | **1** |
| A47 | 0 | 1 | 0 | 0 | 0 | 0 | 0 | 0 | 0 | **1** |
| A48 | 0 | 1 | 0 | 0 | 0 | 0 | 0 | 0 | 0 | **1** |
| A49 | 0 | 1 | 0 | 0 | 0 | 0 | 0 | 0 | 0 | **1** |
| **Total** | **31** | **27** | **25** | **33** | **22** | **16** | **25** | **79** | **16** | **274** |

*Notes*: *Tp1* allele A01 corresponds to *T. parva* alleles identical to the one in the three vaccine strains (Muguga, Serengeti-transformed and Kiambu-5).
