# Supplementary material for: Genetic and antigenic variation of the bovine tick-borne pathogen Theileria parva in the Great Lakes region of Central Africa
Source: Parasit Vectors. 2019 Dec 16;12:588. doi: 10.1186/s13071-019-3848-2 (PMC6915983; doi:10.1186/s13071-019-3848-2)
Supplement: Supplementary file 11 — Additional file 11: Figure S3. Neighbor-joining tree showing phylogenetic relationships among 48 Tp1 gene alleles described in Africa. [file 13071_2019_3848_MOESM11_ESM.docx]

Additional file 11: Figure S3. Neighbor-Joining tree showing phylogenetic relationships among 48 *Tp1* gene alleles described in Africa (A01-A49). *Tp1* gene alleles obtained in the present study are indicated by black diamonds. *Theileria parva* alleles found in cattle with no association with buffalo and in laboratory stocks are coloured in blue and those from buffalo and buffalo-associated cattle are shown in Red. Bootstrap values (>50%) are shown above branches. The *Tp1* homologous sequence of *Theileria annulata* (GenBank accession no. TA17450) was used as outgroup. The number in brackets behind alleles names denote the number of *T. parva* isolates carrying the allele. The frequencies of *Tp1* alleles and their corresponding populations/AEZs are detailed in Additional file 9: Table S7. *Tp1* allele A01 corresponds to isolates identical to the three Muguga cocktail vaccine strains (Muguga, Serengeti-transformed and Kiambu-5).
