# Supplementary material for: Genetic and antigenic variation of the bovine tick-borne pathogen Theileria parva in the Great Lakes region of Central Africa
Source: Parasit Vectors. 2019 Dec 16;12:588. doi: 10.1186/s13071-019-3848-2 (PMC6915983; doi:10.1186/s13071-019-3848-2)
Supplement: Supplementary file 12 — Additional file 12: Figure S4. Phylogenetic tree showing the relationships among concatenated Tp1 and Tp2 nucleotide sequences of 93 T. parva samples from cattle in DRC and Burundi. [file 13071_2019_3848_MOESM12_ESM.docx]

**Additional file 12: Figure S4.** **Phylogenetic tree showing the relationships among concatenated *Tp1* and *Tp2* nucleotide sequences of 93 *T. parva* samples from cattle in DRC and Burundi**. The evolutionary history was constructed using the Neighbor-Joining method with 1000 bootstrap replicates. Bootstrap values (>50%) are shown above branches and indicate the degree of support of each node. The concatenated homologous *Theileria annulata* sequence (GenBank accession no. TA17450 and TA19865 for *Tp1* and *Tp2*, respectively) was used as the outgroup. The codes in brackets behind sample names correspond to their respective concatenated *Tp1* (A) and *Tp2* (a) alleles (Aa). Allele A01a01 corresponds to Muguga and Serengeti-transformed vaccine strains, while allele A02a02 is carried by Kiambu-5 strain. Samples are colour-coded based on their agro-ecological origin (U and Red=DRC AEZ1; W and Green = DRC AEZ2; K and Blue=DRC AEZ3; B and Purple =Burundi AEZ1). Detailed sample characteristics and corresponding gene alleles and protein variants are shown in Additional file 3: Table S3.
